# DNDI-6174, a preclinical candidate for visceral leishmaniasis that targets the cytochrome *bc1* complex

**DOI:** 10.1126/scitranslmed.adh9902

**Published:** 2023-12-13

**Authors:** Stéphanie Braillard, Martine Keenan, Karen J. Breese, Jacob Heppell, Michael Abbott, Rafiqul Islam, David M. Shackleford, Kasiram Katneni, Elly Crighton, Gong Chen, Rahul Patil, Given Lee, Karen L. White, Sandra Carvalho, Richard J. Wall, Giulia Chemi, Fabio Zuccotto, Silvia González, Maria Marco, Julianna Deakyne, David Standing, Gino Brunori, Jonathan J. Lyon, Pablo Castañeda Casado, Isabel Camino, Maria S. Martinez Martinez, Bilal Zulfiqar, Vicky M. Avery, Pim-Bart Feijens, Natascha Van Pelt, An Matheeussen, Sarah Hendrickx, Louis Maes, Guy Caljon, Vanessa Yardley, Susan Wyllie, Susan A. Charman, Eric Chatelain

**Affiliations:** 1 Drugs for Neglected Diseases initiative (DNDi), Chemin Camille-Vidart 15, 1202 Geneva, Switzerland; 2 Epichem Pty Ltd, Perth, Western Australia, Australia; 3 Centre for Drug Candidate Optimisation, Monash Institute of Pharmaceutical Sciences, Monash University, Parkville 3052, Australia; 4 Wellcome Centre for Anti-infectives Research, School of Life Sciences, University of Dundee, Dow Street, Dundee DD1 5EH, United Kingdom; 5 Drug Discovery Unit, Wellcome Centre for Anti-infectives Research, School of Life Sciences, University of Dundee, Dow Street, Dundee DD1 5EH, United Kingdom; 6 Global Health Medicines R&D, GlaxoSmithKline, Tres Cantos, Madrid 28760, Spain; 7 Global Investigative Safety, GSK, Collegeville, United States; 8 Medicine Design, GSK, Stevenage, United Kingdom; 9 Global Investigative Safety, GSK, Ware, United Kingdom; 10 Discovery DMPK, GSK, Tres Cantos, Madrid, Spain; 11 Discovery Biology, Griffith University, Nathan, Queensland, Australia 4111; 12 Laboratory of Microbiology, Parasitology and Hygiene (LMPH), University of Antwerp, Universiteitsplein 1, 2610 Wilrijk, Belgium; 13 Faculty of Infectious and Tropical Diseases, London School of Hygiene and Tropical Medicine, Keppel Street, London WC1E 7HT, United Kingdom

## Abstract

New drugs for visceral leishmaniasis that are safe, low cost and adapted to the field are urgently required. Despite concerted efforts over the last several years, the number of new chemical entities with novel mechanisms of action that are suitable for clinical development remains low. Here, we describe the development of DNDI-6174, an inhibitor of *Leishmania* cytochrome *b* that originated from a phenotypically-identified pyrrolopyrimidine series. This compound fulfills all Target Candidate Profile criteria required for progression into preclinical development. In addition to good metabolic stability and pharmacokinetic properties, DNDI-6174 demonstrates potent *in vitro* activity against a variety of *Leishmania* species and can reduce parasite burden in animal models of infection, with potential for sterile cure. No significant flags were identified in preliminary safety studies, including an exploratory 14-day toxicology study in the rat. DNDI-6174 represents the first cytochrome *b* inhibitor to enter preclinical development for visceral leishmaniasis.

## Introduction

Associated with poverty and classified as a neglected infectious disease, leishmaniasis is one of the diseases targeted in the United Nations Sustainable Development Goals (SDGs) agenda, specifically SDG3.3 (1). Leishmaniasis is a complex vector-borne disease in which parasites are transmitted through the bite of female phlebotomine sandflies. There are more than 20 causative species and various manifestations in different regions of the world. These range from visceral leishmaniasis (VL), also known as kala-azar, a systemic disease that is fatal if left untreated, to cutaneous leishmaniasis (CL). In Asia and East Africa, VL is mostly caused by *Leishmania donovani* and the disease is anthroponotic, whereas it is caused by *L. infantum* in Latin America and the Mediterranean region where the disease is zoonotic, with the dog being the main reservoir. Additionally, painless post-VL skin lesions, (which contribute to continued disease transmission), post kala-azar dermal leishmaniasis (PKDL) and muco-cutaneous leishmaniasis (MCL) occur in some regions of the world (2). Patients with HIV/VL co-infection present a further complicated form of the disease (2). Worldwide, 1 in 7 people live in leishmaniasis-endemic areas with an estimated 1 billion at risk of infection from VL and CL. The disease is highly endemic in the Indian subcontinent and in East Africa, with more than 90% of new cases reported to WHO occurring in 7 countries: Brazil, Ethiopia, India, Kenya, Somalia, South Sudan and Sudan with a high rate of underreporting. Every year, there are between 50,000-90,000 new cases of VL responsible for 20,000-30,000 deaths (2). Currently, the region with the highest burden worldwide is Eastern Africa, with most cases observed in Ethiopia, Kenya, Somalia, Sudan, South Sudan and Uganda.

Historically, antimonial monotherapy (such as sodium stibogluconate, SSG) for 20-30 days has been the mainstay treatment for VL. In the last 15 years, efforts have been made to develop better and safer treatments with liposomal amphotericin B, followed by paromomycin (PM) and miltefosine (MIL), being made available for use. Liposomal amphotericin B is currently considered as the standard treatment for the elimination of VL in India. However, this drug is not as effective in East Africa and pentavalent antimonials remain a component of the primary first-line treatment in that area with significant drawbacks in terms of the parenteral route of administration, length of treatment, toxicity and cost. A single first-line treatment (SSG and PM), which has safety concerns combined with poorly effective second-line treatments that are difficult to administer, are not sufficient for elimination of this disease. The priority is therefore to deliver an anti-parasitic drug that will meet the Target Product Profile (TPP) for VL published by Drugs for Neglected Diseases initiative (DNDi) (3) and hence provide an orally active, safe, effective, short-course and field-adapted treatment for leishmaniasis that would have the potential to revolutionize treatment. If possible, DNDi through its Drug Combination Development Platform (DCDP) will develop a combination therapy based on newly developed New Chemical Entities (NCEs) to achieve short course therapy with satisfactory efficacy having utility for all regions with disease and avoiding the emergence of drug resistance. This would improve and simplify current case management and support elimination efforts. The current pipeline of oral NCEs for VL at the translational stage is unprecedented but the risk of attrition remains. Hence, anticipation of a high rate of attrition in NCE development is a compelling argument to continue efforts to add to the pipeline.

Here we report the discovery of DNDI-6174 and data that support its selection as a preclinical development candidate for VL. DNDI-6174 originated from a potent hit discovered following a phenotypic screening campaign conducted by GSK (4) and was optimized via a medicinal chemistry program focused initially on a related kinetoplastid parasite, *Trypanosoma cruzi* (*T. cruzi*). DNDI-6174 belongs to a novel chemical class of pyrrolopyrimidines and displays pharmacological and physicochemical properties that are consistent with DNDi’s VL Target Candidate Profile (TCP). It targets the Qi site of the *Leishmania* cytochrome *bc1* complex (III) and is the first candidate with this mechanism of action (MoA) to reach the preclinical development stage.

## Results

### Identification and optimization of a promising pyrrolopyrimidine

**Fig. 1:**
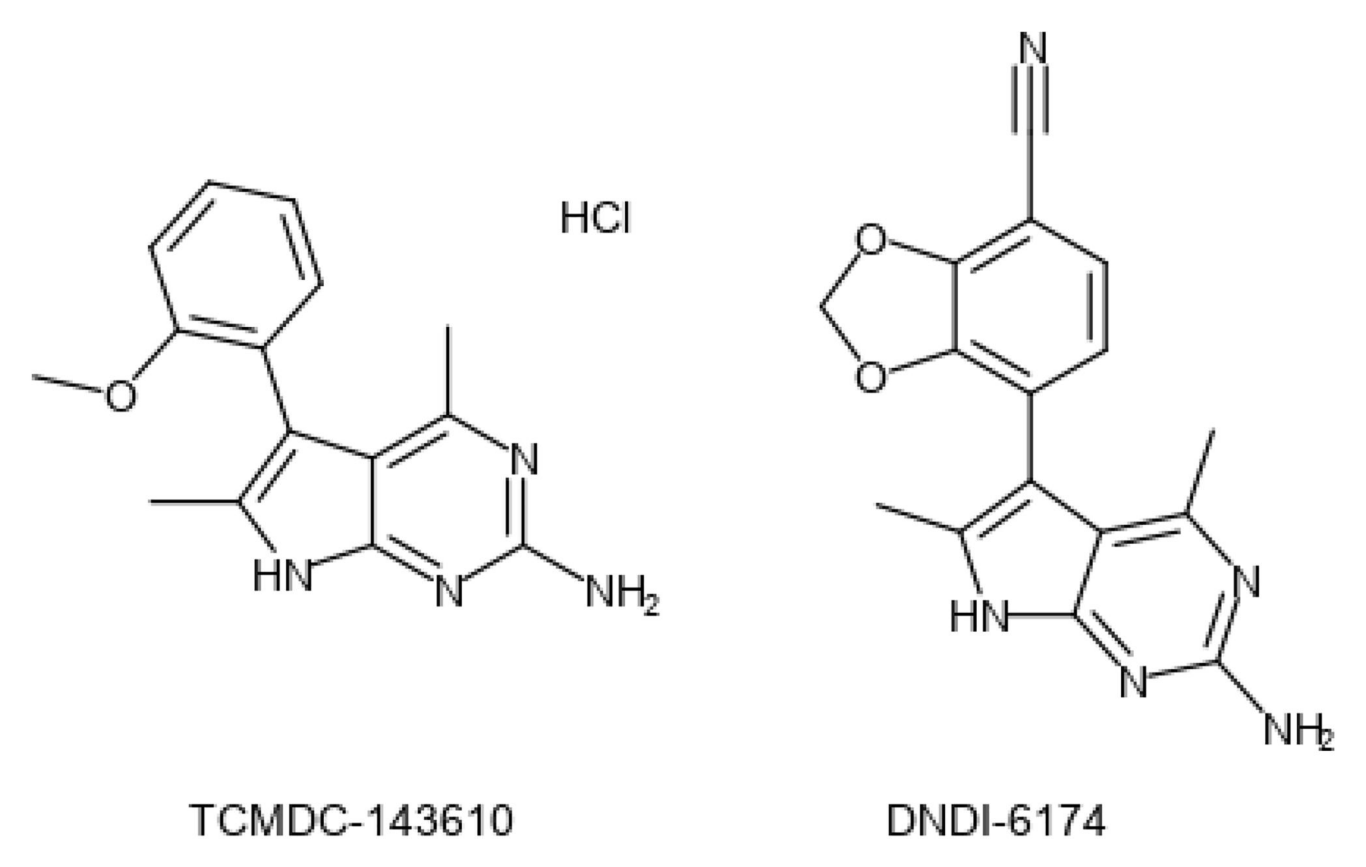
Structures of the initial hit TCMDC-143610 and DNDI-6174

High-throughput screening of GSK’s 1.8M compound library against *L. donovani, T. cruzi* and *T. brucei* resulted in the identification of a significant number of compounds active against these parasites (4). Among these hits, TCMDC-143610 (Fig. 1) demonstrated promising activity against *T. cruzi*, (EC_50_ value of 130 nM), and modest activity against *L. donovani* with an EC_50_ value of 15.8 μM against the intracellular amastigote stage of this parasite. A medicinal chemistry program focused on developing compounds targeting *T. cruzi* successfully delivered potent, advanced leads with excellent pharmacokinetic profiles. Leading compounds were profiled *in vitro* and *in vivo* against *Leishmania spp*. revealing encouraging activity against this parasite as well.

A *Leishmania*-focused lead optimization program was subsequently undertaken to optimize potency, safety, physicochemical and pharmacokinetic properties. The details of the medicinal chemistry program and structure activity relationships (SAR) that led to the discovery of DNDI-0003366174 (aka DNDI-6174) will be described in a separate publication.

### In vitro profiling of DNDI-6174

The potency of DNDI-6174 was established against a broad range of *Leishmania* species and isolates where it was found to be a potent inhibitor of *L. donovani* and *L. infantum*, the etiological agents of VL and was also active against several species that cause CL (Table 1). DNDI-6174 was also found to be broadly active against a panel of representative VL clinical isolates from various sources and importantly, it remained active against clones resistant to currently used therapeutics such as MIL and PM (Table 1).

DNDI-6174 was also profiled for its activity against other disease-causing kinetoplastid parasites. While demonstrating impressive potency against *T. cruzi* (Tulahuen strain, EC_50_ value < 5nM), the causative agent of Chagas disease, the compound was considerably less active against *T. brucei brucei* and *T. b. rhodesiense* (bloodstream form) associated with human African Trypanosomiasis with EC_50_ values ranging from 5 to 20 μM.

### Efficacy of DNDI-6174 in animal models of VL

Given its promising *in vitro* profile, the efficacy of DNDI-6174 was evaluated in murine (acute) and hamster (chronic) models of VL. DNDI-6174 administered for 5 consecutive days at doses of 12.5 mg/kg bid or 25 mg/kg qd resulted in a >98% decrease in the liver parasite burden of infected BALB/c mice compared to control animals (dosed with vehicle). Similar efficacy was observed when the dose was reduced to 6.25 mg/kg bid and the duration of treatment was extended to 10 days. Assessment of 5-day dosing regimens in mice infected with either *L. infantum* or *L. donovani* produced similar results. Indeed, efficacy (>95% reduction in liver parasite burden) was reached at a dose of 12.5 mg/kg bid or higher, while 6.25 mg/kg bid was not sufficient for either species with a 5-day dosing duration. A summary of results obtained following different dose and treatment durations is shown in Table S1A.

To fully appreciate the potential of DNDI-6174 as a drug candidate for VL, the compound was also assessed in a chronic hamster model allowing parasite load evaluation in the target organs: liver, spleen and bone-marrow. Following once daily dosing at and above 12.5 mg/kg showed excellent efficacy, leading to a reduction of the parasite burden of more than 99% in all three organs (Table S1B). The promastigote back-transformation assay was used to demonstrate the potential for sterile cure. Here, the organs of treated animals were recovered, macerated, and introduced to *in vitro* culture (5). Inoculated cultures were then monitored for the emergence of viable promastigotes. In DNDI-6174 treated animals, almost all the organs recovered proved to be negative in culture (Table S2) thus illustrating its potential to effect sterile cure *in vivo*. In contrast, all organs tested in the vehicle control group were positive and more than half of those following miltefosine administration at 40 mg/kg were positive. A once daily administration of 6.25 mg/kg DNDI-6174 was not sufficient for efficacy while bid dosing at 6.25 mg/kg resulted in more than 95% reduction in parasite burden. Consequently, the minimum efficacious daily dose of DNDI-6174 in the hamster model was estimated to be 12.5 mg/kg given qd for 5 days or 6.25 mg/kg given bid for 5 days.

### Mechanism of action studies - cross-resistance profiling

**Fig. 2:**
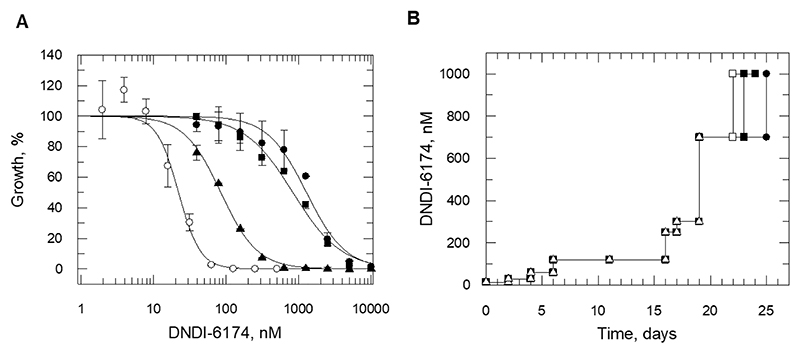
DNDI-6174 targets the Qi site of *L. donovani* cytochrome *b*. (A) Representative EC_50_ curves for DNDI-6174 against WT (open circles) and DDD01716002 Res 1, 2 and 3 (closed circles, squares and triangles, respectively). The curves are the non-linear fits of data using a two-parameter EC_50_ equation provided by GraFit. EC_50_ values of 22 ± 2, 1276 ± 2, 829 ± 6 and 84 ± 3 nM were determined for WT and DDD01716002 Res 1, 2 and 3 lines, respectively. Composite potency data for these cell lines is shown in Table 2. (B) Schematic representation of the generation of DNDI-6174-resistant *L. donovani* lines. Each passage of cells in culture is shown, with clones 1-5 indicated as open circles, closed circles, open squares, closed squares and open triangles, respectively. Note that some points are overlapping and therefore obscured from view. (C) Secondary structure model of the *L. donovani* cytochrome *b* based on the *Saccharomyces cerevisiae* enzyme (8). Amino acids in cytochrome *b* that were mutated in DNDI-6174 resistant lines are indicated by light blue circles.

As a first step towards determining the mechanism of action (MoA), DNDI-6174 was screened against a panel of *L.donovani* promastigote cultures that are resistant to drugs and compounds with defined mechanisms of action and molecular targets. In this screen, three independent clonal cell lines resistant to DDD01716002, an established inhibitor of the cytochrome *bc1* complex of the electron transport chain (ETC) (6), demonstrated considerable (3.4 – 34-fold) cross-resistance to DNDI-6174 (Table 2 and Fig. 2A).

Complex III of the ETC located in the mitochondrial inner membrane is composed of cytochrome *b* associated with a Rieske iron−sulfur protein and cytochrome *c1*. Complex III accepts ubiquinol from complex II of the ETC; the Q_o_ and Q_i_ sites of cytochrome *b* then act in concert to reduce cytochrome *c* by quinone-based electron bifurcation, sequentially oxidizing two ubiquinol molecules to ubiquinone, and then reducing one ubiquinone to ubiquinol (7). DDD01716002 is a specific inhibitor of the Q_i_ site of cytochrome *b* and cell lines resistant to this compound bear mutations within this region of the enzyme (summarized in Table 2).

### MoA studies - resistance generation and whole-genome sequencing

*L. donovani* promastigote cultures resistant to DNDI-6174 were generated by culturing clonal, drug-susceptible parasites in the continuous presence of compound *in vitro* (Fig. 2B). *L. donovani* promastigotes exposed to increasing concentrations of compound over a 25-day period and then cloned by limiting dilution were between 6- and 164-fold less susceptible to DNDI-6174 than the WT parental line (Table 2). In each case the resistance demonstrated by all five of the clones was stable over 20 passages in the absence of compound selection.

Whole genome sequencing (WGS) revealed that all five DNDI-6174-resistant clones-maintained mutations within the gene encoding cytochrome *b* (Fig. 2C and Table 2). All mutations were found to map to the ubiquinone reduction center of cytochrome *b* (Qi site) and notably two were identical to mutations previously identified in DDD01716002-resistant parasites (Gly31Ala and Ser207Pro) (6). Analysis of sequencing indicates that all cytochrome *b* mutations are homozygous. Outside of these mutations, no other common single nucleotide polymorphisms (SNP) or copy number variations (CNV) were identified in the genomes of our drug resistant clones (Table S3, Table S4, and Fig. S1). Cytochrome *b* is encoded solely by the maxi-circle DNA, a minor component of the parasite’s kinetoplast DNA (9), equivalent to mitochondrial DNA in mammalian cells. These mitochondrial DNA networks can maintain up to 50 copies of maxicircle DNA meaning that a single network can encode up to 50 copies of *cytochrome b*. This and other factors effectively preclude investigating the role of specific cytochrome *b* mutations in resistance by genetic methods. Nevertheless, these data strongly suggest that mutations within cytochrome *b* are driving resistance to DNDI-6174 and indicate that cytochrome *b* is the likely target of this promising compound.

#### DNDI-6174 inhibits complex III activity

To establish if DNDI-6174 specifically inhibits complex III activity in *L. donovani*, clarified cell lysates that were enriched in mitochondria were prepared. Complex III activity was monitored in the presence and absence of DNDI-6174 using decylubiquinol as a pseudo-substrate, as previously described (6). As expected, DNDI-6174 proved to be a potent inhibitor of complex III in lysates prepared from both promastigote and axenic amastigote stages of *L. donovani*, returning IC_50_ values of 8 ± 1.7 and 2 ± 0.5 nM, respectively (n ≥ 3 biological replicates). These values correlate with EC_50_ values for DNDI-6174 against promastigotes and axenic amastigotes of 24 ± 1 and 75 ± 2 nM, respectively (n ≥ 3 biological replicates) and support our hypothesis that cytochrome *b* is the principal target of this compound.

#### Modelling

**Fig. 3:**
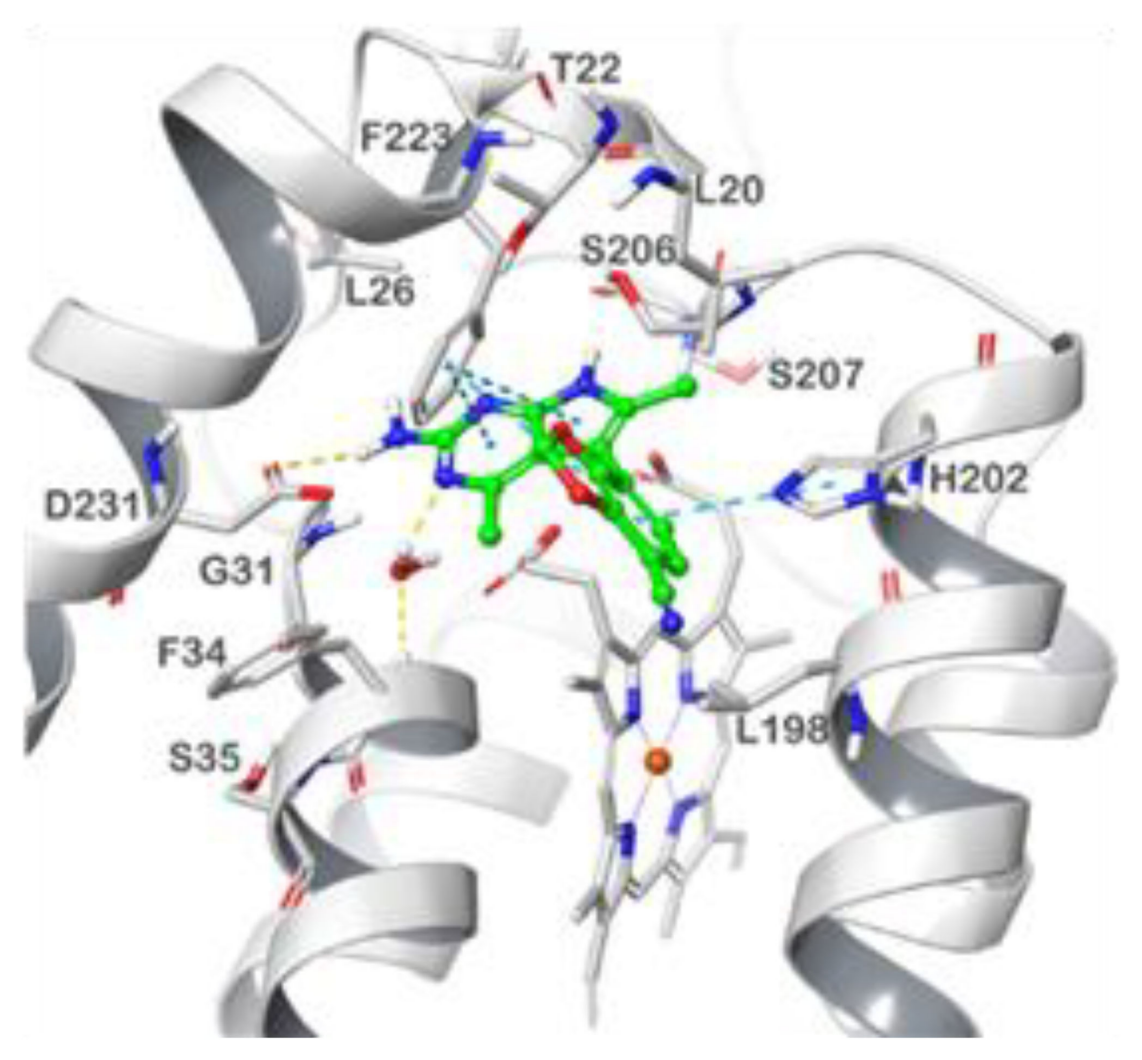
Binding mode of DNDI-6174 in the Qi site of *L. donovani* cytochrome *b* evaluated through molecular docking. Docking mode for DNDI-6174 in wild-type cytochrome *b*. Key binding site residues are highlighted as blue-white sticks, with the heme (b_H_) cofactor and a conserved water molecule also indicated. The hydrogen bond interactions are indicated as yellow dashed lines and the *π*-*π* stackings in cyan.

To gain insight into the binding mode of DNDI-6174 to *L. donovani* cytochrome *b* and to rationalize the role of mutations associated with resistance, we carried out molecular modelling studies. Our previously established *L. donovani* cytochrome *b* model was used as a starting point for these studies (6). In the first instance, DNDI-6174 was docked into the Q_i_ site of WT cytochrome *b*. In the most favorable binding pose, the 2-aminopyrrolopyrimidine scaffold fits well into the proposed binding site and is stabilized by polar interactions with Asp231 and a water-mediated H-bond with the backbone of Phe34 (Fig. 3, S2A-B). Multiple *π*-*π* stackings are established both by the heterocyclic scaffold and the benzodioxole moiety with Phe223 and His202, respectively.

Our *L. donovani* cytochrome *b* model was then used in molecular dynamics simulations at 100 ns. These studies provided a rationale for the impact of resistance conferring mutations. DNDI-6174 was docked into models of the five mutated enzymes. In all cases, DNDI-6174 was accommodated into the Q_i_ binding site of cytochrome *b* in a similar manner to that seen for the WT enzyme, with no major clashes observed (Fig S2). However, these mutations are predicted to impact the binding of DNDI-6174 in several ways, principally by reducing the stability of target-ligand interactions. A detailed description of the effects of individual mutations on ligand binding is provided in supplementary information (Fig. S3-8).

### Physicochemical, permeability and binding properties of DNDI-6174

Physicochemical, permeability and binding properties for DNDI-6174 are shown in Table S5. DNDI-6174 is characterized as having a relatively low molecular weight, moderate lipophilicity and low solubility in physiologically relevant biological media. Binding of DNDI-6174 to plasma proteins from various species is moderate.

The permeability of DNDI-6174 across Caco-2 cell monolayers was found to be high with an efflux ratio of approximately 1.0. The effective human jejunal permeability (P_eff_) was predicted using the measured Caco-2 apparent permeability coefficient (A-B P_app_) and the previously described relationship between Caco-2 permeability and P_eff_ (10) determined using the same experimental conditions. The predicted P_eff_ is high (5.1 x 10^-4^ cm/s) suggesting that permeability is not likely to limit oral absorption in humans. Based on the DCS classification system (11), the solubility limited absorbable dose (SLAD) is approximately 35 mg indicating that solubility within the intestine could limit oral absorption if the human dose is high and formulation approaches may be needed to address this issue.

### Nonclinical metabolism of DNDI-6174

The metabolism of DNDI-6174 was assessed *in vitro* using liver microsomes and cryopreserved hepatocytes from mice, rats, dogs and humans. Intrinsic clearance and predicted *in vivo* hepatic plasma clearance (factoring in binding to the *in vitro* test system, plasma proteins, and the blood to plasma ratio) are shown in Table S6. Overall, microsomes appeared to give better predictions of the *in vivo* clearance in dogs, rats and mice compared to hepatocytes, assuming clearance occurs predominantly by hepatic metabolism (see subsequent pharmacokinetic sections). Based on these predictions, DNDI-6174 would be expected to have low plasma clearance (~1 mL/min/kg) in humans.

**Fig. 4:**
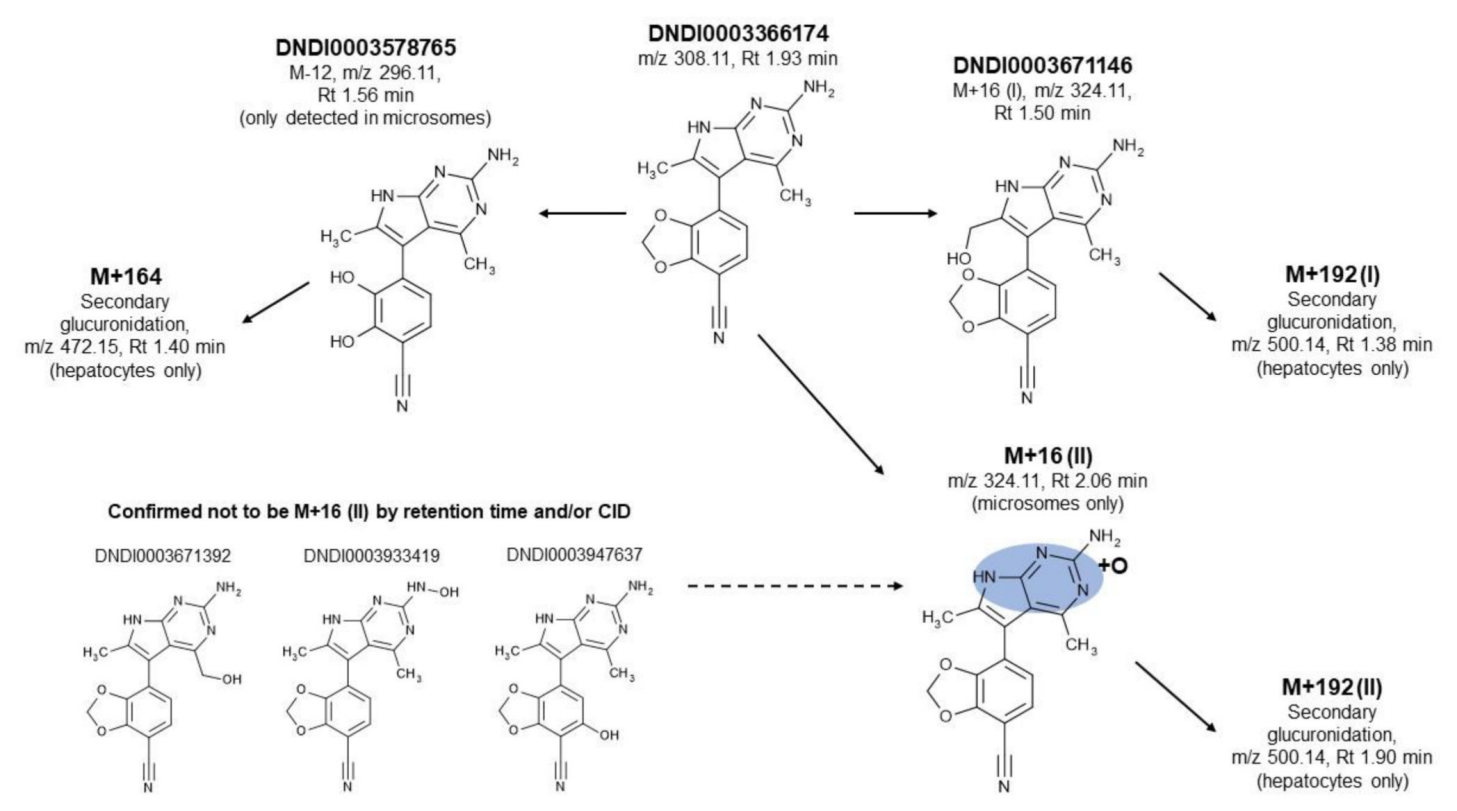
Proposed hepatic metabolism pathways for DNDI-6174 (DNDI0003366174). Data generated *in vitro* following incubation with liver microsomes or cryopreserved hepatocytes.

Further studies were conducted to determine the major metabolites of DNDI-6174 using *in vitro* test systems (liver microsomes and hepatocytes) and comparison of the chromatographic retention and MS/MS fragmentation characteristics to authentic standards for the proposed metabolites. The results are summarized in Fig. 4. In liver microsomes, three primary metabolites were detected, corresponding to two oxygenation products having molecular ions 16 amu higher than the parent compound (designated M+16 (I) and M+16 (II)) and a product with a molecular ion 12 amu lower than the parent (designated M-12). In addition to these three primary metabolites, two secondary metabolites having masses of 34 amu higher than the parent were also detected. Qualitatively, the same metabolites were formed in human, dog and rat microsomes. Two potential metabolites were synthesized (DNDI0003671146 and DNDI0003578765, Fig. 4) and were confirmed to be metabolites M+16 (I) and M-12, respectively; both were found to be inactive against *Leishmania*. Three other potential metabolites were also synthesized (DNDI0003671392, DNDI0003933419 and DNDI0003947637, Fig. 4), however, none of these were detected in either the microsome or hepatocyte incubations. For incubations with hepatocytes, several secondary products were seen that corresponded to glucuronide conjugates of M-12 (designated M+164), of M+16 (I) (designated M+192 (I)) and of M+16 (II) (designated M+192 (II)) (Fig. 4). The identity of M+16 (II) has yet to be confirmed but based on the potential metabolites that can be excluded and CID fragmentation patterns, it is thought to result from *N*-oxygenation of one of the pyrrolopyrimidine nitrogens.

Reaction phenotyping studies with chemical inhibitors were conducted using human liver microsomes to determine the main enzyme(s) involved in the primary metabolism of DNDI-6174. There was no significant difference in the CL_int_ values in the absence or presence of inhibitors for any of the isoforms tested, however, there was a non-significant trend for a reduction in CL_int_ in the presence of CYP1A2 and 3A4/5 inhibitors suggesting a potential contribution of these enzymes (Fig. S9). By monitoring metabolite formation (Fig. S10) it was clear that M+16 (I) is formed by CYP3A4/5 and CYP2B6, and M+16 (II) is formed by CYP1A2. M-12 appeared to be formed by a combination of CYP3A4/5 and CYP2C8. Using the available authentic metabolite standards, only about 75% of the initial DNDI-6174 could be accounted for by the remaining parent and the known primary metabolites suggesting the presence of additional, yet to be identified minor metabolites (e.g. M+16 (II) and possibly others).

### Nonclinical pharmacokinetics of DNDI-6174

**Fig. 5:**
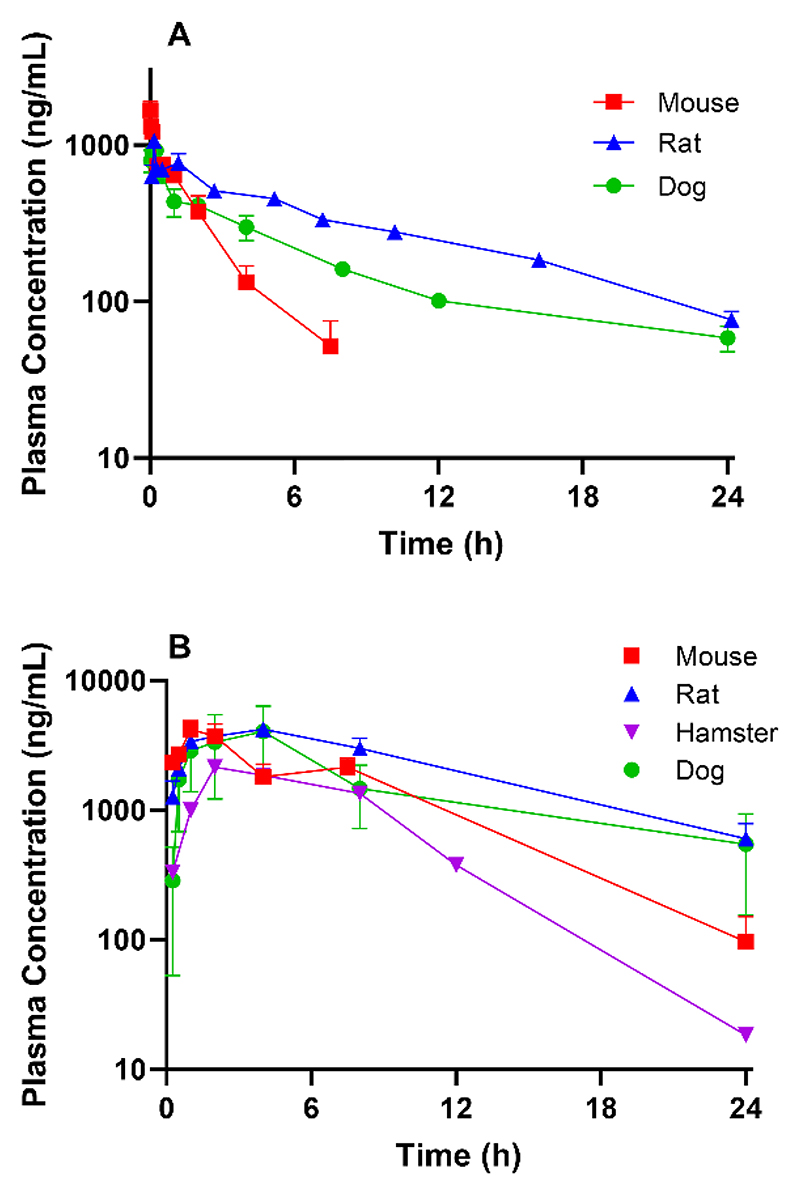
Intravenous (A) and oral (B) plasma concentration versus time profiles for DNDI-6174 following single dosing to mice, rats, hamsters and dogs. IV dosing to hamsters was not conducted. To facilitate species comparisons, the IV data have been normalized to a dose of 1 mg/kg and the oral to a dose of 10 mg/kg, assuming linear kinetics across the dose range. Actual data at the administered doses are shown in Table S7.

The *in vivo* pharmacokinetic properties for DNDI-6174 were characterized in female BALB/c mice, male Sprague Dawley rats, male beagle dogs and female golden Syrian hamsters. Following intravenous administration (Table S7 and Fig. 5), the compound had low plasma clearance, a moderate volume of distribution and moderate half-life across species. In rats, <1% of the parent compound was excreted unchanged in urine, suggesting that hepatic metabolic clearance is the major route of elimination.

Data following single oral administration in mice, rats, dogs and hamsters (day 1 of a multiple dose regimen for hamsters) are also shown in Table S7 and Fig. 5. The oral bioavailability in mice was high and exceeded 100% at 25 mg/kg indicating saturable first-pass and/or systemic clearance. In rats, oral bioavailability was also high and C_max_ and AUC increased in proportion to the increase in dose between 10 and 50 mg/kg (Table S8) but dropped off as doses increased up to 300 mg/kg. In dogs, single dose exposure appeared to increase to a greater extent than the increase in dose between 5 and 30 mg/kg, but then decreased relative to dose from 30 to 90 mg/kg (Table S9). Intravenous dosing in hamsters was not conducted and therefore bioavailability could not be calculated for this species.

Following bid dosing in mice (Figure S11A), a marginal increase in C_max_ (approximately 2-fold) was observed, but AUC (0-24 h and 96-120 h) remained relatively constant over the 5-day dosing period (Table S10). In hamsters (Figures S11B), both C_max_ and AUC increased over the dosing period following qd dosing for 5 days (Table S11).

One-compartmental fits of the mouse concentration versus time data were generally good and the error estimates for the primary parameters were generally less than 20% (Table S12). Error estimates for the secondary parameters were less than 15% at all dose levels. There was more variability in the hamster profiles, which led to greater error in the estimated primary parameters. Error estimates for the secondary parameters were less than 15% at all but the lowest hamster dose.

Fitted data for AUC over 24 h at steady state (AUC_24_ ss) and C_max_ at steady state as a function of dose are shown in Fig. S12 along with the experimental data on days 1 and 5 of dosing. Note that AUC_24_ ss is equivalent to AUC_0-∞_ for a single dose and twice AUC_0-∞_ for twice daily dosing, assuming linear kinetics. On balance, the fits provide a reasonable estimation of the profiles at each dose level and species and were considered suitable for simulating repeat dose profiles under the conditions used in the efficacy studies where exposure could not be assessed for practical reasons.

### PK/PD analysis and human dose prediction

To determine the correlation between the pharmacokinetic (PK) and pharmacodynamic (PD) data, repeat dose profiles in mice and hamsters following single and twice daily dosing were simulated using the fitted parameters. A necessary assumption was made that the PK properties were comparable in healthy and diseased animals. Simulated profiles are shown in Fig. S13. Pharmacodynamic data in mice and hamsters infected with *L. infantum* or *L. donovani* and in hamsters infected with *L. infantum* and treated with DNDI-6174 are tabulated along with the extracted plasma parameters in Table S13 and Table S14, respectively.

**Fig. 6:**
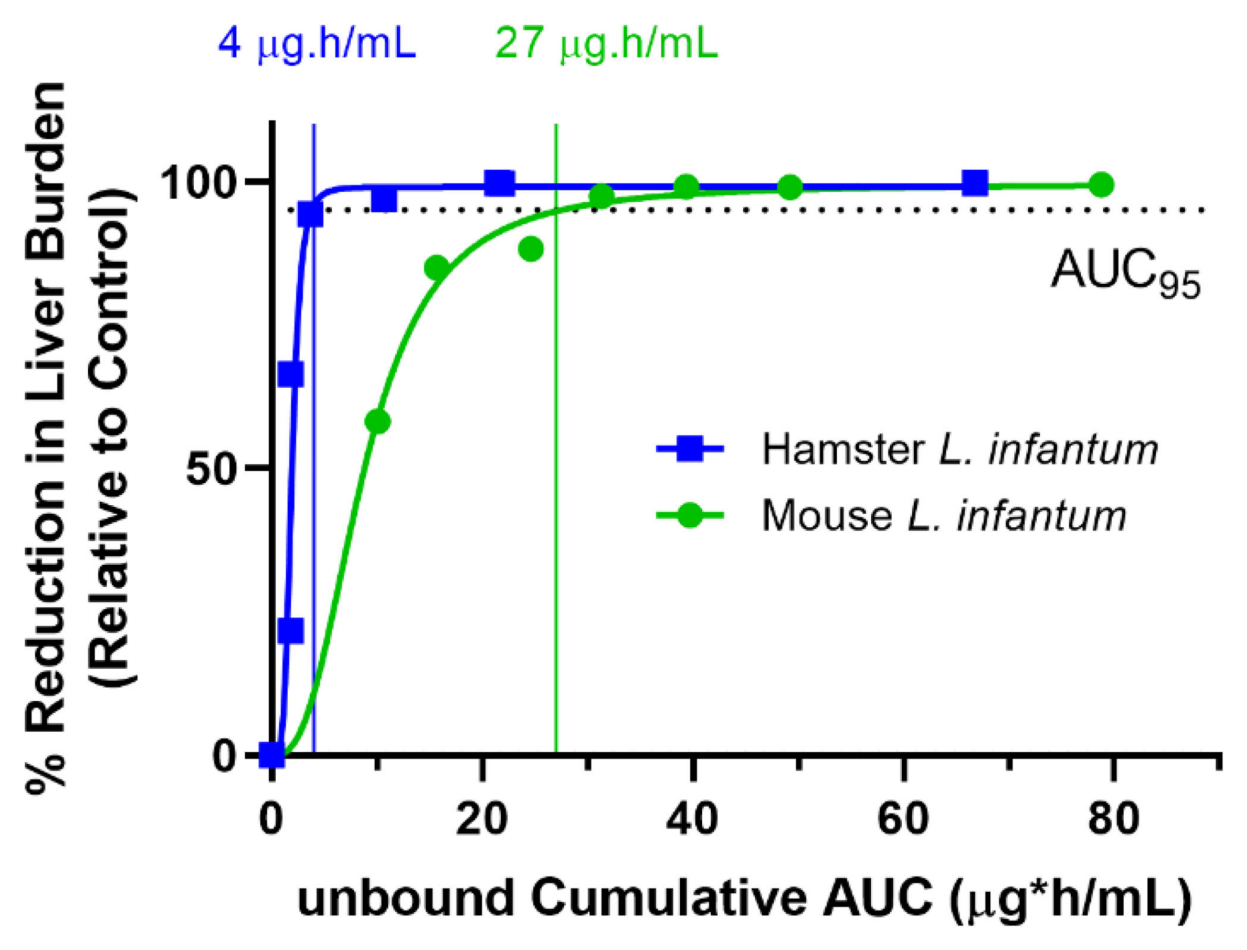
Reduction in liver burden relative to control as a function of cumulative DNDI-6174 unbound AUC in *L. infantum*-infected mice and hamsters. AUC_95_ reflects the cumulative AUC required to achieve greater than 95% reduction in liver parasite burden.

As shown in Fig. 6, higher cumulative exposure (unbound AUC) was required in mice (acute model) compared to hamsters (chronic model) to achieve 95% reduction in liver burden (Table S15). Other parameters were less well correlated to efficacy, presumably given that none reflect the cumulative exposure profile with an extended treatment regimen. Based on these results, the cumulative unbound exposure (AUC) required for 95% reduction in liver burden relative to control is approximately 4 μg.h/mL based on data in hamsters and approximately 27 μg.h/mL based on mice. Results for mice infected with *L. donovani* were similar to those for *L. infantum* (Table S15).

Human plasma clearance was estimated by *in vitro/in vivo* extrapolation (IVIVE) of data from liver microsomes and allometry using the available *in vivo* preclinical data. In both cases, renal excretion of unchanged drug was assumed to be negligible (experimentally confirmed in the rat). For IVIVE, the geometric mean of the error in the predicted unbound intrinsic clearance across the three preclinical species (1.4-fold underprediction) was used as a correction factor for predicting the human unbound intrinsic clearance as described previously (12). A body weight of 50 kg for the patient population was used giving a predicted human plasma clearance of 1.15 mL/min/kg (3.45 L/h). Simple allometry of unbound clearance from mice, rats and dogs was found to be unsuitable for the prediction of human clearance as the allometric exponent was high (>1.0). Therefore, the “rule of exponents” was applied and the unbound clearance data were corrected for brain weight (13) as described previously (14). Using this approach, the predicted human clearance (again assuming a body weight of 50 kg) was 0.90 mL/min/kg (2.71 L/h). The mean of these two values therefore gives a final predicted human clearance of 1.03 mL/min/kg (3.08 L/h)

The predicted clearance was input along with the data in Table S16 into a physiologically-based pharmacokinetic (PBPK) model (GastroPlus) to simulate human profiles under different dosing conditions. Given that there was no evidence of renal elimination in rats, it was assumed that clearance occurs solely by hepatic metabolism. The model predicted half-life and volume of distribution in humans (50 kg body weight) are 12.0 h and 1.1 L/kg, respectively.

Human PK profiles were then simulated to determine the dose and dose regimen necessary to achieve the target exposure profile based on data in hamsters and mice (cumulative unbound plasma AUC of 4 and 27 μg.h/mL, respectively equivalent to a cumulative total human plasma AUC of 60 and 460 μg.h/mL, Fig. S14). For a 10-day dosing period, the estimated human dose is 20-180 mg (qd) or 10-80 mg (bid). For a 14-day dosing period, the estimated dose is 15-120 mg (qd) or 7.5-55 mg (bid).

### Early safety profiling of DNDI-6174

*In vitro* de-risking was performed to identify any potential cardiotoxicity, cytotoxicity and mutagenicity liabilities that could have precluded the clinical development of DNDI-6174. *In silico* and *in vitro* risk assessment of cardiotoxicity predicted little to no change in QT interval at exposures up to 30 μM but a profile possibly associated with an effect on hemodynamics at high exposures (Table S17 and Fig. S15). In cytotoxicity assays, DNDI-6174 showed no activity (CC_50_ >37 μM) against all cell lines tested (Table S18). The AMES analysis of DNDI-6174 did not show intrinsic mutagenic potential in the absence or presence of rat liver S9 fraction for metabolic activation. In mammalian test systems (mouse lymphoma and CHO micronucleus), again in the absence or presence of rat liver S9, DNDI-6174 was not genotoxic.

DNDI-6714 did not show any activity in panels of mammalian receptor, enzyme or ion channel assays (Table S19), with the exception of alpha1A Human Adrenoceptor GPCR (α1A antagonism) and the human phosphodiesterases PDE3A and PDE4D2 for which an IC_50_ values of 12.8, 3.36 and 6.67 μM, respectively, were measured. These activities observed for PDEs were not considered as relevant as physiological levels of these enzymes are at the low nM range (concentrations of cAMP, cGMP).

Since DNDI-6174 targets the electron transport chain in the mitochondria of *Leishmania*, a significant selectivity towards human cytochrome *bc1* and no mitochondrial toxicity were desirable. The assessment of DNDI-6174 for potential mitochondrial toxicity followed a 3-step cascade whereby specific criteria were defined in order to continue development of this compound (Table S20). A significant window (>1000 fold) was observed between the human cytochrome bc1 complex biochemical assay (<20% inhibition at 200 μM) and the effects on the bc1 complex of *Leishmania donovani*. In the MitoExpress^®^ mitochondrial function assay, oxygen consumption was reduced at concentrations ≥66 μM in HepG2 cells, with a maximum response limited to 28% at the highest concertation tested (200 μM). Likewise in the Seahorse^®^ mitochondrial stress test in HepG2 cells, only a small reduction in oxygen consumption rate (OCR Basal) was observed up to a maximum of 14% reduction at 200 μM; the minimum effect concentration (MEC) for OCR reduction was 95.6 μM and there were no changes in Reserve Capacity, ECAR, Maximum Capacity or ATP production. Compared to the free concentration of DNDI-6174 at the predicted therapeutic plasma C_max_ in humans, this provides approximately 57-fold to the MitoExpress^®^ no-effect concentration in HepG2 cells and approximately 187-fold to the Seahorse^®^ OCR MEC. There was a reduction in the calcium loading capacity of isolated mitochondria with DNDI-6174 treatment (XC50 = 10 μM), suggesting potential for some mitochondrial activity at lower concentrations that warrants further consideration, however the overall pattern of mitochondrial data with DNDI-6174 does not suggest significant direct inhibition of mammalian cytochrome bc1 complex.

The risk of DNDI-6174 being phototoxic was discarded according to the results generated in the *in vitro* 3T3 Neutral Red Uptake (NRU) phototoxicity assay.

An exploratory 14-day toxicology study was performed with DNDI-6174 at a daily dose of 30, 80 or 200 mg/kg. All rats tolerated oral administration of 30 and 80 mg/kg with no mortality or morbidity. The dose of 200 mg/kg led to early sacrifice of one female on day 5. Furthermore, most animals of this group showed clinical signs suggesting a lack of tolerability including a body weight decrease of >10% in males which correlated with a decrease in food consumption. At this highest dose, white blood cell count was also affected and associated with decreases in thymus and spleen weights, consistent with stress. Minimal increase in liver enzyme markers (alanine aminotransferase and aspartate aminotransferase) and total bilirubin concentration occurred in males at 80 and 200 mg/kg/day, while increased liver weights occurred in females at 80 and 200 mg/kg/day. Higher thyroid/parathyroid gland weights were observed in males at and above 80 mg/kg/day. In the absence of adverse findings at 80 mg/kg, this dose has been set as the no observed adverse effect level (NOAEL) for DNDI-6174. The dose of 80 mg/kg in the rat resulted in a 9-to 33-fold higher exposure (AUC_0-24h_ at steady state of 756 and 865 μg.h/mL in males and females, respectively) compared to the steady state AUC needed in the mouse or hamster for efficacy.

### Early drug-drug interaction risk assessment

DNDI-6174 exhibited moderate inhibition against CYP1A2 (IC_50_ of 5.1 μM) and CYP3A4/5 (IC_50_ of 12.3 μM) and minimal inhibition (IC_50_ >15 μM) of the remaining isoforms (Table S21). Experiments conducted with a pre-incubation in the absence and presence of the co-factor, NADPH, suggested time-dependent inhibition of CYP2D6 and CYP3A4/5, and to a lesser degree CYP2C8.

DNDI-6174 was also assessed against a panel of drug transporters at concentrations of 1 and 10 μM showing no significant inhibition (>50% activity remaining) of BCRP, BSEP, OAT1, OAT3 OCT1 and OCT2. Low but significant inhibition was observed on OATP1B1 (IC_50_ = 22 μM) and OATP1B3 (IC_50_ = 16 μM), while substantial inhibition of MATE2-K (IC_50_ = 0.88 μM) and MATE1-HEK (IC_50_ = 2.1 nM) was measured.

## Discussion

Following a phenotypic screening campaign conducted by GSK (4), we have optimized a series of pyrrolopyrimidines as a new chemical class for potential treatment of leishmaniasis and identified DNDI-6174 as a promising oral preclinical candidate. DNDI-6174 showed potent *in vitro* antileishmanial activity against a range of *Leishmania* species responsible for causing VL or CL, as well as current drug resistant species and clinical isolates. While this compund was found to be very potent against another parasite of the Kinetoplastida, *T. cruzi*, DNDI-6174 was only marginally active against *T. brucei*.

Comprehensive MoA studies confirmed that DNDI-6174 employs a different mechanism of action compared to anti-leishmanials in clinical use as well as the other NCEs in the current global leishmaniasis portfolio, an important consideration if one envisages to combining drugs for a better therapeutic outcome This pyrrolopyrimidine inhibits *Leishmania* cytochrome *b*, a component of complex III (cytochrome *bc1*) of the parasite’s electron transport chain. Specifically, DNDI-6174 interacts with the Q_i_ active site of this mitochondrial enzyme. While cytochrome *b* was identified through chemical genomics as a possible target for new drug discovery efforts aimed at treating Chagas disease (15), recent studies have shown that the Q_i_ site of cytochrome *b* is a promiscuous drug target in *L. donovani* and *T. cruzi* (6). Drugs targeting cytochrome *b* are in clinical use for treatment of malaria and fungal pneumonia, and cytochrome *b* was also reported as a promising target for treatment of tuberculosis (16–18). DNDI-6174 is the first compound with this MoA to move forward into development for VL.

DNDI-6174’s *in vitro* antiparasitic potency combined with good metabolic stability across species allowed its testing in different *in vivo* models of *Leishmania* infection. Its assessment in both *L. donovani/L. infantum* mouse and hamster models exhibited very high levels of efficacy in all organs at doses as low as 12.5 mg/kg for 5 days or 6.25 mg/kg for 10 days, leading to a reduction of the parasite burden of more than 99% in all organs with a potential for sterile cure. Further characterization indicated that despite only moderate solubility, DNDI-6174 showed good permeability and had excellent pharmacokinetic properties (although possible solubility limited absorption and bioavailability at high dose); PK/PD analysis suggested a predicted curative dose in humans ranging between 20-180 mg (qd) or 10-80 mg (bid) for a 10-day dosing period. This predicted human dose regimen, despite the broad dose range, is very favorable and compares well, or is even lower than the Standard of Care and other NCEs currently in development.

Given its target, potential mitochondrial toxicity of DNDI-6174 was a concern and a specific screening cascade with clear Go/NoGo criteria was developed. In the biochemical assay against the mammalian cytochrome *bc1* complex a therapeutic window above 1000 was determined. In the functional HepG2 cell-based MitoExpress^®^ and Seahorse^®^ assays mitochondrial activity with DNDI-6174 was found to be limited. A reduction in the calcium loading capacity of isolated mitochondria was observed suggesting the potential for some mito-activity with DNDI-6174 which warrants further consideration, however the overall pattern across all the endpoints was not consistent with a significant inhibition of the mammalian cytochrome bc1 complex. Early receptor and enzyme profiling did not show any relevant flags and a generally good *in vitro* safety profile was observed. Moreover, DNDI-6174 did not show any phototoxicity, genotoxicity, or safety pharmacology flags, and an encouraging therapeutic index following a 14-day exploratory toxicity study in the rat was achieved.

Overall, DNDI-6174 combines impressive preclinical *in vivo* efficacy with appropriate pharmaceutical properties and an acceptable safety profile fulfilling all DNDi’s TCP criteria for VL. In addition, it shows potential for CL, as it demonstrated similar levels of *in vitro* activity against species of *Leishmania* responsible for that disease. This makes DNDI-6174 suitable for onward development against all forms of leishmaniasis.

The results are limited by the not yet fully established translation of the *in vivo* models of the disease and consequently by an understanding of what is the most adequate model for human dose prediction. This is reflected by the large dose range for the predicted human dose due to differences in the efficacy data depending on the species used and the animal model considered (mouse or hamster). Clinical data will provide an indication of the value of each of these models, and which one is more appropriate for use in the future human dose predictions.

Another potential issue is linked to the molecular target of DNDI-6174 itself. Cytochrome *b* is encoded by kinetoplastid DNA, equivalent to mitochondrial DNA in other eukaryotes. Replication of kinetoplast DNA is considered error prone and thus genes encoded by the kinetoplast are associated with a particularly high mutation rate which may lead to a higher resistance potential. However, it should be noted that malaria parasites bearing mutations in cytochrome *b* and resistant to atovaquone are not transmissible by mosquitoes (19). The apparent loss of fitness of atovaquone-resistant parasites in the mosquito has been associated with the higher respiratory rate required at this stage of the lifecycle. The failure of these mutated parasites to be transmitted effectively limits the spread of atovaquone resistance in the field. It remains to be seen if a similar fitness cost will be associated with *L. donovani* parasites bearing Q_i_ site mutations. Regardless, future treatment strategies for VL are focusing on the development of combination therapies to limit the development of resistance to treatment. In that respect, and in the context of development of a combination therapy, more work will be necessary to assess in more detail the potential risk of drug-drug-interactions with DNDI-6174 given its potential time-dependent inhibition of CYP2D6 and CYP3A4/5, and to a lesser degree CYP2C8 observed.

Overall, the data package for DNDI-6174 supports its continued development to determine whether this compound can become a much-needed safe oral treatment for patients suffering from multiple forms of the devastating neglected tropical disease leishmaniasis. It is currently under preclinical development with the goal of starting clinical Phase I in 2023.

## Materials and Methods

### Ethical statements

All animal experiments were performed according to institutional ethical guidelines for animal care. More precisely, Sprague Dawley rats and BALB/c mice PK studies were conducted at Monash University according to protocols reviewed and approved by the University’s Animal Ethics Committee and in accordance with the Australian Code of Practice for the Care and Use of Animals for Scientific Purposes. Sprague Dawley rats and Beagle dog PK studies conducted at WuXi were in accordance with institutional and national guidelines at WuXi AppTec (the Institutional Animal Care and Use Committee (IACUC)). Efficacy hamster and mouse studies at LMPH were carried out in strict accordance with all mandatory guidelines (EU directives, including the Revised Directive 2010/63/EU on the protection of Animals used for Scientific Purposes that came into force on 01/01/2013, and the declaration of Helsinki in its latest version) and were approved by the ethical committee of the University of Antwerp, Belgium (UA-ECD 2011-74 and 2019-10). The efficacy mouse model study at LSHTM was carried out under a UK Home Office project license according to the Animal (Scientific Procedures) Act 1986 and the new European Directive 2010/63/EU. The project license (70/8427) was reviewed by the LSHTM Animal Welfare and Ethical Review Board prior to submission and consequent approval by the UK Home Office. The design of the Wistar rat toxicity study conducted at Charles River Laboratories France Safety Assessment SAS (AAALAC accredited test Facility) was reviewed and approved by the ethical committee of the Test Facility as per the standard project authorization no. 2017072617402851. The study design was in general compliance with the following animal health and welfare guidelines: Guide for the care and use of laboratory animals (2011), Decree n° 2013-118 relating to the protection of animals used in scientific experiments described in the Journal Officiel de la République Française on 01 February 2013, Directive 2010/63/EU of the European Parliament and of the Council of 22 September 2010 on the protection of animals used for scientific purposes.

### Leishmania intramacrophage in vitro assay

Primary peritoneal macrophage (PMM) host cells were infected at a ratio of 1:5 with *L. infantum* MHOM/MA(BE)/67/ITMAP263 spleen-derived amastigotes isolated from heavily infected donor hamsters or at a ratio of 1:15 with late stationary-phase promastigotes of the different VL and CL strains (Table 1). A drug incubation period of 96 h was applied, and cells were stained with Giemsa for microscopic evaluation of cellular amastigote burdens. Percentage reduction compared to the burden in the infected non-treated control wells was used as a measure for drug activity.

Alternatively, differentiated macrophage host cells derived from THP-1 (human leukemia monocytes) were infected at a multiplicity of infection of 1:2.5 (ratio of host cells to parasites) with *L. donovani* MHOM/IN/80/DD8 culture-maintained promastigotes. *L. donovani* MHOM/IN/80/DD8 (ATCC 50212) promastigote parasites and THP-1 (ATCC TIB202) cells were maintained as previously described (20). A drug incubation period of 96 h was applied, plates fixed with 4% paraformaldehyde and stained with SYBR^®^ green and CellMask Deep Red™ (ThermoFisher Scientific). Images were acquired on an Opera high-content imaging system (PerkinElmer). Healthy host (THP-1) cells and intracellular amastigotes were identified using CellMask Deep Red cytoplasmic and SYBR green nuclear area and intensities, with intracellular parasites identified based on spot detection algorithms (size and intensity measurements used to define parasite nucleus) to determine the number of parasites present within THP-1 host cells. An infected cell was defined as a host cell containing >3 parasites within the cytoplasm boundary. Compound activity was determined based on the number of infected cells normalized to the positive (10μM DNDI-1044) and negative (0.4% DMSO) controls. Non-linear sigmoidal dose-response curves with no constraints were plotted, and IC_50_ was calculated. The IC_50_s were calculated from two independent experiments.

### In vivo efficacy studies in mice and hamsters

#### Acute mouse infection model (VL)

Female 8−10-week-old BALB/c mice were infected with 2 x 10^7^ *L. donovani* MHOM/ET/67/L82 (synonym HU3) or *L. infantum* MHOM/MA67/ITMAP263 amastigotes on day 0 and dispatched in groups of five individuals. On day 6 post-infection, treatment was initiated by oral gavage once a day for 5 or 10 days. Five days following end of treatment, all mice were sacrificed, and parasite burden was determined from smears of liver sections. Efficacy was expressed as the mean percentage load reduction compared to untreated (vehicle-only) control animals. Reference drugs miltefosine and/or AmBisome were used as positive controls and comparators.

#### Chronic hamster infection model (VL)

Female golden hamsters (5 per treatment group) were infected through intracardiac administration of 2x10^7^ *L. infantum* amastigotes obtained from the spleen of a heavily infected donor hamster. DNDI-6174, miltefosine as reference control or vehicle were orally administrated from day 21 post-infection. After 5-day treatment period, followed by a 10-day wash out period, animals were sacrificed, and parasites burden assessed in the three target organs (liver, spleen, and bone-marrow). For this assessment, organs of individual animals were weighed, except for bone-marrow thus only providing a semi-quantitative evaluation only. Impression smears were Giemsa-stained for microscopic evaluation of amastigote burden, expressed as LDU (= mean number of amastigotes/cell × organ weight in mg) and the results were expressed as a percentage reduction compared to the burdens in the control group (vehicle as placebo). For evaluating the presence of viable residual burden after treatment, a promastigote back-transformation assay was conducted. This consisted of the incubation at ambient temperature of aseptically collected pieces of spleen or liver tissue in 1 mL of promastigote back-transformation medium in 24-well plates. For the bone-marrow, resected femurs were flushed with 1 mL medium. The medium consists of HOMEM pH 6.0 prepared as described elsewhere (5) with 10% fetal bovine serum (Gibco), 2.5% penicillin/streptomycin and 1% gentamicin (Merck). A qualitative assessment of the presence of promastigotes was made after 3 and 5 days of incubation and a score was attributed (+, ++ or +++) based on parasite density in the positive wells.

### Human complex III inhibition assay

Preparation of mitochondria from a human cell line (THP-1) was performed following published procedure (21). The protocol for the assay has been described in (22).

### Calcium loading capacity (CLC) assessment in HEK293F-derived mitochondria

The CLC of frozen HEK293F-derived mitochondria was determined using a method based on that previously described for fresh rat liver mitochondria (23). Briefly, a buffer (130 mM Sucrose, 37.54 mM KCl, 2.5 mM KH_2_PO_4_, 5 mM HEPES) containing 0.5 mM Ca^2+^ and supplemented with 15 mM succinate, 2.5 μM rotenone and 0.15 μg/ml cell impermeant Fluo 5N (Life Technologies), was added to each well of a 384 well microplate (control wells also contained 0.24 μl/well DMSO or 3 μM cyclosporine). Thawed mitochondria suspended at 0.6 mg/ml were added to all wells to initiate the assay, and the fluorescence measured every 5 minutes at 520 nm in a PHERAstar FS microplate reader (BMG LabTech, Aylesbury, UK), (25 reads over 2 hours). Area under the curve (AUC) values for the first 10 reads were exported and the concentration-response relationship was modelled using the following formula: y=((B-A)/(1+(10^X/10^C)^D)+A where B=max, A=min, C=IC50, D=slope. The fitted curves were then used to calculate a pXC_50_ value.

### Assessment of Mitochondrial Function Using the MitoXpress Assay

The effect of DNDI-6174 on oxygen consumption was assessed using the MitoXpress Oxygen Consumption Assay according to the manufacturer’s instructions. Briefly, HepG2 cells were seeded in 96-well plates in 100 μL of complete media and incubated overnight in an incubator set to maintain approximately (37°C and 5% CO_2_). The cells were then washed once and then fresh media was added containing the MitoXpress^®^ Probe and DNDI-6174 at final assay concentrations of 0 (0.5% DMSO, vehicle control) 2.5, 7.4, 29, 66.7 and 200 μM. Oligomycin (5 μM), FCCP (1 μM), or antimycin A or antimycin A/Rotenone (1 μM) were added to additional wells as positive controls. Prewarmed (37°C) 100% mineral oil (100 μL) was immediately added to all wells. The assay plates were transferred to a Perkin Elmer Wallac VICTOR3 System reader for time-resolved fluorescence measurements. Fluorescence (excitation 340 nm/emission 642 nm) was measured every two minutes for approximately 1-2 hrs (or until signal plateau/saturation is reached).

The dual-read time-resolved (T1 = 30 μs and T2 = 70 μs) fluorescence intensity readings (R1 and R2) from the Perkin Elmer Wallac VICTOR3 system (Perkin Elmer) were converted to lifetime fluorescence values as per guidelines detailed in the Agilent MitoXpress Xtra Oxygen Consumption Assay Manual. Calculation of the slope translates to oxygen consumption rate (OCR). Oxygen consumption rate was multiplied by 60 to determine the slope/hour for each assay well. Average slope values for each treatment were normalized to the average slope value for Control/Vehicle Only (0 uM DNDI-6174, 0.5% DMSO) treatment. A t-test analysis (unequal variance, unpaired, 2-tailed) was used to compare each compound concentration of DNDi-6174 against the Control/Vehicle Only (0 uM DNDI-6174, 0.5% DMSO) treatment.

### Assessment of Mitochondrial Function Using the Seahorse^®^ Assay

Seahorse^®^ 96 well plates were seeded with HepG2 cells and incubated overnight (37°C, 5% CO_2_). The media in all plate wells was then replaced with Seahorse^®^ assay media and the plates were incubated (37°C, CO_2_ free) for a further 1 hour before placing in the Seahorse^®^ XFe96 analyser (Agilent Technologies Inc, USA). Following calibration, four baseline oxygen consumption rate (OCR) and extracellular acidification rate (ECAR) measurements were made and then DNDI-6174, vehicle control or rotenone control, previously prepared in Seahorse^®^ assay media, were injected into the plate wells. OCR and ECAR were measured for 45 minutes before the sequential addition of oligomycin (ATP synthase inhibitor), FCCP (carbonyl cyanide 4-(trifluoromethoxy) phenylhydrazone, protonophore uncoupler) and rotenone plus antimycin A (Electron transport chain Complex I and III inhibitors respectively). Multiple measurements of OCR and ECAR were made following each of these additions.

The Seahorse^®^ data for DNDI-6174 treated cells were expressed as a ratio of the mean DMSO vehicle control data for the plate. Mean ± standard deviation of the compound ratio data were plotted vs log concentration and curves fitted (as described in 24) and the minimum effective concentration (MEC) determined using limits based on the DMSO vehicle mean ± 2 standard deviations or, where these limits were deemed too narrow, the DMSO vehicle mean ± minimum limits set for each parameter. The AC_50_ was calculated using the curve and historical maximum (activation) and minimum (inhibition) responses and is defined as the concentration at which 50% of the maximum or minimum effect is observed, provided a clear concentration response relationship is seen.

### Cell lines and culture conditions (mode of action studies)

The clonal *L. donovani* cell line LdBOB (derived from MHOM/SD/62/1S-CL2D) was grown as promastigotes at 28°C and as axenic amastigotes at 37°C, as described previously (25).

### Drug sensitivity assays (mode of action studies)

To examine the effects of test compounds on growth in 96-well plates, promastigote or axenic amastigote cultures seeded at 5 × 10^4^ parasites mL^–1^ were incubated in the presence of 2-fold serial dilutions of test compounds for 72 h. Following incubation, 50 μM resazurin was added to each well and fluorescence (excitation of 528 nm and emission of 590 nm) measured after a further 3 h incubation. Data were processed using GRAFIT (Erithacus software) and fitted to a 2-parameter equation, where the data are corrected for background fluorescence, to obtain the effective concentration inhibiting growth by 50% (EC_50_):

[1] $$y=\frac{100}{1+{\left(\frac{\left[I\right]}{EC_{50}}\right)}^{m}}$$

In this equation [*I*] represents inhibitor concentration and *m* is the slope factor. Experiments were repeated at least two times and the data is presented as the mean plus standard deviation.

### Generation of compound-resistant parasites

Compound-resistant lines were generated by subculturing clones of WT *L. donovani* in the continuous presence of DNDI-6174. Starting at sublethal concentrations, drug concentrations in 5 independent cultures were increased in a stepwise manner, usually by 2-fold. When parasites were able to survive and grow in 1 μM DNDI-6174, the resulting lines were cloned by limiting dilution in the presence of compound. Five clones (Res 1–5) were selected for further biological study.

### Whole genome sequencing

Genomic DNA was collected from wild type and resistant line *Leishmania donovani* promastigotes and sequenced by the Beijing Genomics Institute (BGI). Sequence reads were aligned to the *L. donovani* BPK282A1 genome (v39, tritrypDB) with maxi-circle (CP022652.1, NCBI) as described previously (6). Median read counts of the wild type and resistant lines were used to normalise copy number. The associated data sets have been deposited with the European Nucleotide Archive under the following accession number: PRJEB45584.

### Complex III assays

Measurement of complex III activity and inhibition were performed as described previously (6).

### Molecular modelling

#### Ligand and enzyme preparation

The 3D model of DNDI-6174 was created using LigPrep (Schrödinger Release 2021-2, LigPrep, Schrödinger, LLC, New York, NY, 2021), generating the most probable ionization state and tautomers at neutral pH (7.4 ± 0.2). Our previously reported homology model of *L. donovani* cytochrome *b* (6) was processed using the protein preparation module in the Schrödinger suite to provide starting points for subsequent molecular modelling studies. New structures were generated incorporating each of the five mutations found to drive drug resistance. For every mutant, the hydrogen atoms’ positions were optimized using the H-bond assignment/sample water orientation tool and the resulting structures were subjected to a restrained minimisation procedure with the OPLS3e force field. This was achieved using the minimisation protocol of the protein preparation module of the Schrödinger’s suite of software.

#### Molecular docking

Molecular docking was performed using the six cytochrome *b* structures (wild-type and mutated) with ubiquinone bound in the Q_i_ site alongside one conserved water. Molecular docking studies were carried out using Glide (Schrödinger Release 2021-2, Glide, Schrödinger, LLC, New York, NY, 2021) in standard precision (SP). The docking energy grids for wild-type and mutant cytochrome *b* were prepared using the default value of the protein atom scaling factor (1.0 Å) within a cubic box centered on the ubiquinone (UQ2) bound in the wild-type model. The conserved water molecule interacting with Phe34 was also retained. After grid generation, DNDI-6174 was docked into cytochrome *b*. The number of poses entered to post-docking minimization was set to 10. In order to assess the validity of the docking protocol, UQ2 was used as a reference ligand for a redocking procedure with all 6 enzymes (WT and 5 mutants).

#### Molecular dynamics

DNDI-6174: cytochrome *b* complexes generated during molecular docking studies underwent 100 ns molecular dynamic (MD) simulations using Desmond (Schrödinger Release 2021-2: Desmond Molecular Dynamics System, D. E. Shaw Research, New York, NY, 2021. Maestro-Desmond Interoperability Tools, Schrödinger, New York, NY, 2021). The C-terminal carboxylic acid of all model proteins was capped with N-methylamine, and the N-terminal position acetylated. Bilayers of 1-palmitoyl-2-oleoyl-sn-glycero-3-phosphocholine (POPC) membranes were added to the model systems to mimic the integral membrane environment of cytochrome *b*. The obtained systems were placed in orthorhombic boxes filled with simple point-charge (SPC) water molecules. To ensure an electrically neutral system for the simulation, counter ions were added to the system in the form of a 0.15 M salt solution. The ions were placed 10 Å away from the ligands as a buffer zone. Prior to running simulations, the systems were allowed to relax. The NPγT ensemble class was chosen, so that fixing the surface tension ensured that the simulation box does not deform significantly in the plane of the membrane while the pressure is applied normal to the membrane surface. The analysis of all the MD trajectories was performed within the Schrödinger’s simulation interactions diagram panel.

### Physicochemical properties and Caco-2 permeability

The ionization constant (pKa), octanol-pH 7.4 buffer partition coefficient (Log D_7.4_), solubility in biorelevant buffers (FaSSIF-V2, FeSSIF-V2, FaSSGF and pH 7.4 phosphate buffered saline (PBS)) and Caco-2 permeability were determined according to previously published methods (10).

### Protein binding

Human plasma was purchased from Innovative Research, Inc (Novi, MI, USA). Dog (beagle), rat (Sprague Dawley) and hamster (golden Syrian) plasma were procured from Valley Biomedical Products and Services, Inc. (Winchester, VA, USA). Mouse (BALB/c) plasma was collected in-house (animals sourced from Monash Animal Services, Monash University). All plasma was stored frozen at -20°C until use.

Plasma protein binding was assessed using rapid equilibrium dialysis (RED) according to methods published previously (10). To minimize the potential for non-specific adsorption to the dialysis apparatus and to decrease the time needed for equilibration, RED inserts were pre-saturated to a dilute solution of DNDI-6174 in PBS for 24 h prior to the dialysis experiment and the pre-saturation solutions were then discarded. Dialysis was performed by spiking diluted plasma (10% in PBS) with DNDI-6174 and dialysing against PBS for 24 h at 37°C on an orbital plate shaker (800 rpm, ThermoMixer C, Eppendorf). Parallel spiked aliquots of 10% plasma were used to assess compound stability under the same incubation conditions. At the completion of the dialysis period, aliquots were taken from the donor and dialysate chambers to obtain measurements of the total and free concentration, respectively. The pH of the matrix and dialysate were confirmed to be within pH 7.4 ± 0.1 for the duration of the experiment.

Binding to liver microsomes was conducted in a similar manner as described for plasma by spiking human liver microsomes (1 mg/mL) prepared in 0.1 M phosphate buffer (pH 7.4) with DNDI-6174 (0.5 μM), equilibrating for approximately 10 min at 37°C, and then adding to RED units. Dialysis was conducted for 6 h and parallel non-dialyzed samples were included to confirm the absence of compound degradation. Binding to hepatocytes (at a cell density of 1 x 10^6^ cells/mL) was calculated from the measured microsomal binding (at 1 mg/mL) as described previously (26). The fraction unbound (f_u_) was determined from the ratio of the dialysate to the donor concentration, assuming that the system was at steady state at the end of the dialysis period. For plasma, the data were corrected for the dilution factor to give a binding value for neat plasma according to previously published methods (27).

### Blood to plasma partitioning

Whole blood to plasma partitioning (B:P) was determined in human, rat and mouse whole blood and blood stability was assessed in human and rat whole blood. Fresh dog blood was not available,e so the average of the other three species was used as an estimate. For rat and human, aliquots of whole blood were spiked with DNDI-6174 to a concentration of 1000 ng/mL and maintained at 37°C under a 5% CO_2_ atmosphere to maintain the pH at 7.4 ± 0.1. At 30 and 120 min, eight aliquots of the whole blood were transferred to fresh microcentrifuge tubes and four were used for stability assessment and to determine the whole blood concentration, with the remaining four being centrifuged for separation of plasma to determine the blood to plasma ratio. For mouse, blood was collected from mice at 15 min and 1 h after an IV dose of 3 mg/kg into tubes containing heparin, samples were gently mixed before removing aliquots of whole blood and centrifugation of the remaining blood for the collection of plasma. For analysis, blood and plasma samples were matrix matched by mixing the sample with an equal volume of the opposite blank matrix (i.e. blood samples were mixed with blank plasma and plasma samples were mixed with blank blood) and then quantitated by LCMS against a calibration curve prepared in a 1:1 mixture of blank blood and plasma.

### In vitro metabolism

The *in vitro* intrinsic clearance of DNDI-6174 was determined in human, dog, rat and mouse liver microsomes (Sekisui XenoTech, LLC, Kansas City, KS) at a substrate concentration of 0.5 μM and a protein concentration of 1 mg/mL. Compounds were spiked into microsomal matrix prepared in 0.1 M pH 7.4 phosphate buffer containing magnesium chloride (final concentration of 3.3 mM), equilibrated for 5-10 min at 37°C. The reaction was initiated with the addition of freshly prepared solution of NADPH (final 1.3 mM). Control samples without co-factor were included for comparison. Aliquots of the reaction mixtures were taken periodically over 60 min and quenched with acetonitrile containing internal standards (metolazone and diazepam). Quenched samples were maintained on ice for approximately 20-30 min, centrifuged (for 5 min) and the supernatant was analyzed by LC-MS.

The *in vitro* intrinsic clearance of DNDI-6174 was also assessed in human, dog, rat and mouse cryopreserved hepatocytes (Sekisui XenoTech) suspended in Krebs-Henseleit buffer at 0.5 μM substrate and a cell density of 1x10^6^ viable cells/mL. Cell viability was determined using trypan blue exclusion. The incubation was performed on a plate shaker (Heidolph Titrimax 100, 900 rpm) placed in a humidified incubator (37°C, 7.5% CO_2_) for 240 min. The reaction was initiated with the addition of compound with samples taken periodically and quenched with the addition of acetonitrile containing internal standards (metolazone and diazepam). Quenched samples were maintained on ice for approximately 20-30 min, centrifuged (for 5 min) and the supernatant analyzed by LC-MS.

The LC-MS conditions for both microsomal and hepatocyte samples consisted of a Waters Xevo G2-S QToF coupled to a Waters Acquity UPLC, an Ascentis Express C8 column (50 x 2.1 mm, 2.7 μm) and a mobile phase consisting of water and acetonitrile, each containing 0.05% formic acid and delivered under gradient conditions over 6 min. The injection volume was 3 μL and the flow rate was 0.4 mL/min. MS was conducted in positive mode electrospray ionization under MS^E^ acquisition mode to allow simultaneous acquisition of MS spectra at low and high collision energies.

Metabolite detection was conducted using Waters UNIFI software and metabolites were identified using a combination of accurate mass, MS/MS (CID) spectral analysis and comparison of retention times and CID spectra to those of authentic metabolite standards where available.

*In vitro* intrinsic clearance (CL_int_, μL/min/mg or μL/min/10^6^ cells) values were calculated from the apparent first order degradation rate constant divided by the microsome concentration or the hepatocyte cell number (10^6^ viable cells/mL). *In vitro* CL_int_ values were then divided by the fraction unbound in the *in vitro* test system (to give CL_int,u_) and scaled to *in vivo* values (mL/min/kg) using published physiological scaling factors (28). Blood clearance in each species was then calculated using the well-stirred model of hepatic extraction (equation 2) where Q is the hepatic blood flow and f_u,blood_ is calculated from f_u,plasma_/B:P:

[2] $$\;Predicted\;CL_{blood\;}=\frac{Q\;x\;f_{u,blood}\;x\;CL_{\mathrm{int},u}}{Q+f_{u,blood}\;x\;CL_{\mathrm{int},u}}$$

Blood clearance values were converted to plasma clearance by multiplying by the B:P.

### Reaction phenotyping

Reaction phenotyping was conducted using chemical inhibitors of seven major CYP isoforms: CYP1A2 (125 μM furafylline), CYP2B6 (75 μM 2-phenyl-2-(1-piperidinyl) propane (PPP)), CYP2C8 (10 μM montelukast), CYP2C9 (4 μM sulfaphenazole), CYP2C19 (15 μM (S)-N-3-benzylnirvanol), CYP2D6 (2.0 μM quinidine) and CYP3A4/5 (1.5 μM ketoconazole) as described previously (29). Human liver microsomes (1 mg/mL) prepared in 0.1 M phosphate buffer (pH 7.4) with the addition of MgCl_2_ (3.3 mM) were preincubated for 15 min with NADPH (1.3 mM) in the absence or presence of chemical inhibitors prior to the addition of DNDI-6174 (1 μM). Other aspects of the incubation and analysis were as described above.

### In vivo pharmacokinetic properties

Single dose intravenous (IV) pharmacokinetic (PK) studies in male Sprague Dawley rats and IV and oral PK studies in female BALB/c mice were conducted at Monash University. DNDI-6174 was administered by IV infusion (n=3 rats) over 10 min via a cannula implanted in the jugular vein on the day prior to dosing at a dose of 3 mg/kg prepared in 0.9% saline containing 5% DMSO and 2% Solutol HS-15 (1 mL dose volume). Blood was sampled via a cannula implanted in the carotid artery (also on the day prior to dosing) at pre-determined time points up to 24 h post-dose. Samples were transferred to vials containing heparin as an anticoagulant, gently mixed and centrifuged, and plasma separated for subsequent quantitative analysis using LC-MS. For mice, DNDI-6174 was administered by IV bolus injection into the lateral tail vein (2 mL/kg) at a dose of 2 mg/kg using the same IV formulation vehicle as for rats. A dose of 25 mg/kg prepared in an aqueous suspension vehicle containing 0.5% hydroxypropyl methylcellulose, 0.5% benzyl alcohol and 0.4% polysorbate 80 (0.2 mL per mouse) was also dosed via oral gavage. Mouse blood was sampled via submandibular bleed up to 24 h post-dose (n=2 mice per time point, n=3 samples per mouse) into vials containing heparin and plasma was separated for quantitative analysis as described above.

### Rat, mouse, hamster and dog pharmacokinetic studies

Additional PK studies were also conducted by WuXi AppTec Co, Ltd (Shanghai, China) according to protocols reviewed and approved by DNDi. Studies were conducted in male Sprague Dawley rats (IV dose of 3 mg/kg and single oral doses of 10, 50, 100 and 300 mg/kg), female BALB/c mice (twice daily (at 6 and 24 h) oral doses of 3.13, 6.25 and 12.5 mg/kg for 5 days), golden Syrian hamsters (single oral dose of 50 mg/kg and once daily oral doses of 6.25, 12.5 and 25 mg/kg for 5 days) and in beagle dogs (IV dose of 1 mg/kg and single oral doses of 5 and 30 mg/kg). IV and oral formulations for rats, mice and hamsters were the same as described above. For dogs, the IV dose was administered in saline containing 2% DMSO and 3% Solutol HS-15 and the oral formulation was as described above. Blood was sampled up to 48 h post-dose into vials containing potassium EDTA as an anticoagulant, proteins were precipitated with acetonitrile and plasma was separated and assayed by LC-MS.

### Bioanalytical methods

At Monash University, plasma samples were assayed by LC-MS/MS following protein precipitation and quantitated against calibration standards prepared in blank matrix. Briefly, proteins were precipitated from plasma samples by the addition of acetonitrile (3:1 volume ratio) containing diazepam as an internal standard after which samples were centrifuged and the supernatant injected onto the LC-MS system. Urine samples were treated with an equal volume of acetonitrile and assayed against calibration standards prepared in 50% acetonitrile/water with diazepam as the internal standard. The LC-MS system consisted of a Waters Acquity UPLC coupled to either a Waters Xevo TQS Micro or Waters Xevo TQ mass spectrometer. Chromatography was conducted using a Supelco Ascentis Express RP Amide column (50 x 2.1 mm, 2.7 μm) maintained at 40°C and a mobile phase consisting of phase A (water containing 0.05% v/v formic acid) and B (acetonitrile containing 0.05% v/v formic acid) and mixed under gradient elution conditions (4 min cycle, 0.4 mL/min flow rate). MS conditions included ESI positive mode with multiple reaction monitoring using a cone and CID voltages of 50 V and 35 V, respectively. The elution of DNDI-6174 was monitored using a transition (m/z) of 308.17 > 280.17. The calibration range was from 1 to 10,000 ng/mL and the lower limit of quantitation was 1 ng/mL. Precision (% relative standard deviation) and accuracy (% bias) were <5% and within ± 15%, respectively.

For studies conducted at WuXi, plasma samples were assayed using a similar procedure as described above with the exception of the column (Acquity UPLC BEH C18 column (2.1 x 50 mm, 1.7 μm) maintained at 60°C) and mobile phase (phase A: 95% water (containing 0.1% formic acid and 2 mM ammonium formate)/5% acetonitrile; phase B: 95% acetonitrile (containing 0.1% formic acid and 2 mM ammonium formate)/5% water) mixed under gradient conditions over a 4 min cycle with a flow rate of 0.6 mL/min.

### Pharmacokinetic data analysis

Single dose pharmacokinetic parameters (clearance, volume of distribution and half-life) were initially estimated using non-compartmental methods and oral bioavailability was calculated using the ratio of the dose corrected AUC after oral dosing relative to that after IV administration. Nominal doses were used in the calculations unless the measured dose differed by more than 15% from the nominal dose, in which case the measured dose was used. Where a full concentration versus time profile was obtained from a single animal, parameters were estimated for each animal and mean and standard deviations calculated. In the case of mice where sparse sampling was used with multiple animals per time point, the data analysis was conducted using the mean profile.

To support pharmacokinetic/pharmacodynamic (PKPD) analysis, data in mice and hamsters were also analyzed by compartmental modeling using Phoenix WinNonlin (version 8.2; Certara, Princeton, NJ). For each dose level, an extravascular dosing, single compartment disposition model was fit to the combined Day 1/Day 5 repeat dose plasma data. The model was parameterized with a single volume term (V/F), and with first order absorption (K01) and elimination (K10) rate constants. Initial values for the parameter estimates were obtained by fitting of the model to the Day 1 data at the lowest dose level only, and the same initial values were used across the dose range. Experimental data used in model fitting were the mean concentration-time profiles and the average dose administered to each group. The weighting scheme (1/Y2 for mouse and 1/Y for hamster) was selected based on goodness of fit of the peak region of the experimental profiles. Parameter estimates obtained through fitting were used to simulate full repeat-dose plasma concentration-time profiles with both once-daily and twice-daily (dosing at 8 and 24 h) administration at each dose level and treatment duration used in the efficacy studies.

Human clearance was estimated using *in vitro/in vivo* extrapolation (IVIVE) of data obtained in liver microsomes and cryopreserved hepatocytes incorporating binding to microsomes and hepatocytes, plasma protein binding and the blood to plasma ratio. Scaling factors were as described previously (12) and liver blood flows were from Simcyp, assuming an average human body weight of 50 kg was assumed for the patient population (12). A correction factor was calculated based on the geometric average fold-error in unbound intrinsic clearance across the preclinical species (1.4-fold underprediction for microsomes) as described previously (12), and this value was used to correct the predicted human unbound intrinsic clearance. Allometry of unbound clearance was also used, however a brain weight (13) correction was applied given that the exponent for simple allometry was >1.0 (14).

The human dose predictions were based on PK and PD data in hamsters (best case scenario) and mice (worst case scenario) and unbound plasma concentrations that led to 95% efficacy. Human profiles were simulated at different doses and dosing regimens (qd or bid) over 10-14 days using GastroPlus (version 9.8, Simulations Plus, Inc., Lancaster, CA). Input parameters are shown in Table S16. The PBPK model used fasted physiological conditions and a 50 kg 30-year-old American male. The Berezhkovskiy perfusion limited model was used for Kp estimation (30). The estimated human plasma clearance was input directly into the model with the assumption that elimination occurred only by hepatic metabolism (consistent with data in rats).

### AP-preDICT simulation

ApPredict is a simulation engine, a bolt-on extension to the software package Chaste, to perform simulations of drug-induced changes to the cardiac action potential (https://chaste.cs.ox.ac.uk/trac/wiki/ApPredict). This *in silico* model of human and rabbit cardiac myocyte allows prediction of the action potential response to ion channel modulation.

### hERG channel test on QPatch^HTX^

The effects of DNDI-6174 on Chinese hamster ovary cells stably expressing hERG potassium channels was assessed by WuXi AppTec (Shanghai) Co., Ltd., using the automated patch clamp method. Two concentrations (1 and 30 μM, two replicates each) were tested at room temperature.

### hiPSc-CM multielectrode (MEA) assay

The hIPSc-CM assay was conducted to identify DNDI-6174-induced changes in field potential duration (FPD ≈ QT interval), total spike amplitude (TSA ≈ QRS interval) and beat rate. Inducible pluripotent stem cell-derived cardiomyocytes (hiPSc-CMs, iCell2, Cellular Dynamics) were maintained in culture medium (50,000 cells/well) for 7 days. Full media changes were done every 48 h. Following baseline recording, and right after the 1 hour compound treatment time (n=8/concentration, 5 concentrations), 1 min recording of activity was taken daily starting on Day 3 through Day 7 using the Maestro multi-electrode Array System (Axion BioSystems; 48 or 96-well plate). 3μM Quinidine was used as a control for hERG and hNaV1.5 block.

### In vitro cytotoxicity assays

Cytotoxicity profiling of DNDI-6174 was evaluated as previously described using primary mouse macrophages (PMM) and human fetal lung fibroblasts (MRC5) (31), and 3T3 (32), HepG2 (33), HFF (34), THP1 (35), U2OS (36) and Vero cells (37).

### Receptor, enzyme and ion channel and drug transporter assays

DNDI-6174 profiling was conducted at 10 μM concentration in an *in vitro* pharmacology panel at Eurofins (https://www.discoverx.com/home). Listed values represent the percent change relative to the DMSO control activity. When an IC_50_ had to be determined, eight different concentrations were included. All values are the mean of assay readout duplicates.

### Ames test

DNDI-6174 (at concentration levels of 50, 150, 500, 1500 and 2500 μg/plate) was evaluated in the Ames reverse mutation test both in the presence and absence of metabolic activation (rat liver S9). *Salmonella typhimurium* strains TA1535, TA1537, TA98 and TA100, and *Escherichia coli* WP2 uvrA (pKM101) were employed. Positive controls were respectively sodium azide (2 μg/plate), 9-amino acridine (50 μg/plate), 2-nitrofluorene (1 μg/plate), sodium azide (2 μg/plate) and 4-nitroquinoline-1-oxide (2 μg/plate). The negative control was DMSO.

### In vitro micronucleus assay in CHO-K1 cells

DNDI-6174 (at concentrations from 1000 to 0.152 μM) was assessed by WuXi AppTec Co., Ltd. (Shanghai), in the *in vitro* micronucleus assay (96-well plates) using the CHO-K1 cell line and based on OECD (Test No. 487) guidelines (38). The treatment time was 24 h without metabolic activation (rat liver S9) and 3 h with activation. The positive controls were bleomycin sulfate and cyclophosphamide, respectively. 1% DMSO was used as negative control and genotoxic potential was estimated by evaluating the ability of DNDI-6174 to induce micronucleus formation.

### In vitro mutation assay with L5178Y mouse lymphoma cells at the TK locus

DNDI-6174 (at concentrations of 5, 10, 20, 80 and 100 μg/mL) was assessed in the *in vitro* mouse lymphoma mutation assay cells to assess its potential to induce non-lethal gene mutations and chromosome damage in L5178Y (TK+/-) mouse lymphoma cells (39, 40). Two independent mutation tests were carried out: one in which cells were treated for 3 h in the presence of rat liver S9 mix and with methyl methanesulphonate as a positive control, and a second treated for 24 h in the absence of S9 mix and with dimethylbenzanthracene as a positive control. In both cases, DMSO was used as the negative control. In accordance with current guidelines ICH S2(R1), 2011 (41) mutant frequency was counted at concentrations where solubility was not a limiting factor and cytotoxicity was not observed.

### In vitro phototoxicity test

Phototoxicity testing was conducted by WuXi AppTec Co., Ltd. (Shanghai), and absorption peaks for DNDI-6174 were measured by Nanodrop1000 at 242 and 318 nm and were thus found to be within the range of natural sunlight (290-700 nm –λmax 255 nm). Extinction coefficients were 16131 and 8507 L mol^-1^ cm^-1^, respectively. An *in vitro* 3T3 Neutral Red Uptake (NRU) phototoxicity assay was then conducted using a 96-well cytotoxicity-based assay that utilizes normal BALB/c 3T3 mouse fibroblasts to measure the concentration-dependent reduction in neutral red uptake by the cells after exposure to test material either in the presence or absence of UVA light. The assay was performed according to OECD (Test No. 432) test guidance (42). Duplicate 96-well monolayers of 3T3 fibroblasts were exposed to serial dilutions of test material. One of the plates was exposed to 5 J/cm2 UVA while the other plate was kept in the dark. To assess viability, the NRU by cells exposed to the test chemical in the presence of UVA exposure was compared to the NRU by cells exposed to the test chemical in the absence of UVA exposure. The Photo-Irritation-Factor (PIF) and Mean Photo Effect (MPE) were calculated.

### Rat toxicology study

The 14-day toxicology study with DNDI-6174 was conducted by Charles River Laboratories France Safety Assessment SAS. DNDI-6174 was prepared in 0.5% (w/v) hydroxypropyl methylcellulose, 0.5% (v/v) benzyl alcohol and 0.4% (v/v) Polysorbate 80 in Milli-Q water and orally administrated by daily gavage at dose levels of 30, 80 and 200 mg/kg to groups of 22 Wistar Han rats (5 main and 6 satellite animals per sex) for 14 days. A control group of 16 rats (5 main and 3 satellite animals per sex) received a similar volume (5 mL/kg) of the vehicle alone. Parameters monitored included morbidity/mortality, clinical signs, body weight, food consumption, hematology and serum clinical chemistry. Main study animals were killed on the day after the last dose and necropsied. Selected organs were weighed. Organ/tissue samples were fixed and preserved at necropsy. Following the macroscopic examination, selected organs/tissues were examined histopathologically and compared to the control group. Satellite animals were sampled for toxicokinetics on days 1 and 14, at various time-points after treatment.

### Cytochrome P450 inhibition

Direct CYP inhibition studies were conducted using conditions described previously (43) following incubation of isoform-specific substrates with human liver microsomes. The formation of known metabolites mediated by a specific CYP isoform was monitored. CYP isoform-specific pathways (and positive control inhibitors) included CYP1A2: phenacetin *O*-deethylation (furafylline), CYP2B6: bupropion hydroxylation (PPP), CYP2C8: amodiaquine deethylation (quercetin) CYP2C9: tolbutamide methylhydroxylation (sulfaphenazole), CYP2C19: (S)-mephenytoin 4’-hydroxylation (ticlopidine), CYP2D6: dextromethorphan *O*-demethylation (quinidine), and CYP3A: midazolam 1’-hydroxylation and testosterone 6β-hydroxylation (ketoconazole). Briefly, DNDI-6174 (or positive control inhibitor) was incubated (37°C) at varying concentrations (up to 20 μM) in a 96-well plate with human liver microsomes suspended in buffer with addition of each substrate. The organic solvent content was maintained at less than 1% (v/v). NADPH was added to initiate the reaction which was then quenched by the addition of acetonitrile containing internal standard at specified times. Concentrations of the isoform-specific metabolites were quantified by LC-MS (Waters Xevo TQD triple quadrupole MS coupled to a Waters Acquity UPLC system) relative to calibration standards prepared in pre-quenched microsomal matrix. The percent inhibition of metabolite formation was plotted against the log of the inhibitor concentration and IC_50_ values were estimated by fitting data to a 4-parameter sigmoidal function with minimum and maximum inhibition values constrained to 0 and 100%, respectively.

Time dependent inhibition was assessed using an “IC_50_ shift” method based on a previous publication (44). Briefly, DNDI-6174 or positive control inhibitor were preincubated with human liver microsomes at 10-fold the final protein and inhibitor concentration in both the absence or presence of NADPH. Following the preincubation (for 30 min at 37°C) period, mixtures were diluted 10-fold and probe substrates added to determine enzyme activity and IC_50_ as described for the direct inhibition assay. A shift in the IC_50_ in the presence of NADPH (relative to the absence) was used as an indication of time dependent inhibition. Reference direct and time-dependent inhibitors were included in the assays.

## Supplementary Material

Supplementary Materials

## Figures and Tables

**Table 1:** *In vitro* activity (EC_50_ and EC_90_) of DNDI-6174 against different *Leishmania* species/strains.

	Species and strains	EC_50_ (nM)	EC_90_ (nM)
	*L. donovani *MHOM/IN/80/DD8	170	490
	*L. donovani *MHOM/ET/67/HU3	90	2000
	*L. donovani *MHOM/NP/03/BPK275/0-cl18	40	120
	*L. donovani *MHOM/ NP/2003/ BPK282/0-cl4	50	140
VL strains	*L. donovani *MHOM/ET/2007/LLM-1600	80	280
	*L. infantum *MHOM/MA/67/ITMAP263	160	440
	*L. infantum *MHOM/BR/2007/WC (L3015)	70	180
	*L. infantum *MHOM/ES/2016/LLM-2346	210	1520
	*L. infantum *MHOM/FR/96/LEM3323	50	180
	*L. infantum *MHOM/FR/96/LEM3323 Cl4 MIL-R*	70	220
	*L. infantum *MHOM/FR/96/LEM3323 Cl4 PM-R*	80	170
	*L. major *MHOM/SA/85/JISH118	740	11400
	*L. major *MHOM/IL/81/Friedlin	90	900
CL Strains	*L. guyanensis *MHOM/PE/02/PER 054/0	12	39
	*L. tropica *MHOM/AF/2015/HTD7	360	2180
	*L. panamensis *MHOM/PA/71/LS94	40	85

EC_50_ values represent the weighted mean ± standard deviation of at least three biological replicates with each biological replicate comprised of two technical replicates.

* see additional information in the supplementary section.

**Table 2:** Potency of DNDI-6174 against wild-type *L. donovani* (WT), DDD01716002- and DNDI-6174-resistant *L. donovani* promastigote cultures.

*L. donovani *promastigote cultures	EC_50_ values, nM	Fold-change versus WT	Cytochrome *b *mutation
Wild-type	24 ± 1	-	-
DDD01716002 Res 1	779 ± 98	32	S207P
DDD01716002 Res 2	829 ± 63	34	G31A
DDD01716002 Res 3	84 ± 3	3.5	F227I
DNDI-6174 Res 1	431 ± 16	18	S35N/S206N
DNDI-6174 Res 2	3946 ± 228	164	D231E
DNDI-6174 Res 3	1500 ± 91	62.5	S207P
DNDI-6174 Res 4	2699 ± 145	112	G31A
DNDI-6174 Res 5	157 ± 5	6	S206N

EC_50_ values represent the weighted mean ± standard deviation of at least three biological replicates with each biological replicate comprised of two technical replicates.

## Data Availability

All data are available in the main text or the supplementary materials.

## One sentence summary

Here, we report the development of the first inhibitor of *Leishmania* cytochrome *b* with the required properties to enter preclinical development for the neglected tropical disease visceral leishmaniasis.
