# Supplementary material for: DNDI-6174, a preclinical candidate for visceral leishmaniasis that targets the cytochrome bc1 complex
Source: Sci Transl Med. Author manuscript; Available in PMC 2024 Feb 29. (PMC7615677; doi:10.1126/scitranslmed.adh9902)
Supplement: Supplementary Materials [file EMS194242-supplement-Supplementary_Materials.pdf]

Supplementary Materials for

DNDI-6174, a preclinical candidate for visceral leishmaniasis that targets the cytochrome *bc1* complex

This document includes:

Material and methods
Supplementary information on Tables

Fig. S1 Chromosomal CNV relative to wild-type in DNDI-6174-resistant clones.

Fig. S2 Binding mode of DNDI-6174 in the Qi sites of mutated versions of cytochrome *b* (A-E). Ser35Asn and Ser206Asn mutations identified in cell line Res 1 are reported in magenta and green. Mutation Asp231Glu identified in Res 2 is represented in orange. The Ser207Pro mutation identified in Res 3 is in light blue. Gly31Ala (Res 4) and Ser206Asn (Res 5) are reported in wheat and green, respectively.

Fig. S3 (A) The Ligand Root Mean Square Fluctuation (RMSF) for each atom of DNDI-6174 in cytochrome *b* (wild-type) measuring the changes in the position of ligand atoms during the 100 ns MD simulation (atom number as reported in the chemical representation on the right). These studies indicate that benzodioxole moiety of DNDI-6174 is the portion of the molecule with the highest flexibility during binding. (B) A schematic of detailed ligand atom interactions with the amino acid residues of cytochrome *b*. (C) Root Mean Square Deviation (RMSD) plot of DNDI-6174 (right Y-axis – magenta curve) and wild-type cytochrome *b* (left Y-axis – blue curve). The plot indicates the stability of the protein and the ligand during the simulation. The overall stability of the docking binding pose is highlighted by the protein and ligand RMSD over this simulation. (D) Protein-Ligand Contacts plot. The possible interactions are categorized into four types: hydrogen bonds, hydrophobic, ionic and water bridges. The stacked bar charts are normalized over the course of the trajectory.

Fig. S4 (A) The Ligand Root Mean Square Fluctuation (RMSF) of DNDI-6174 during the 100 ns MD simulation in the mutated cytochrome *b* (Ser35Asn/Ser206Asn) from Res 1. Ligand flexibility in this mutated enzyme increased considerably, even for the pyrrolopyrimidine scaffold that was particularly tightly bound in the wild-type enzyme. RMSF is reported by atom number as reported in the chemical representation on the right. (B) A schematic of detailed ligand atom interactions with the protein residues. (C) Root Mean Square Deviation (RMSD) plot of DNDI-6174 and cytochrome b. (D) Protein-Ligand Contacts plot. The possible interactions are categorized into four types: hydrogen bonds, hydrophobic, ionic and water bridges. The stacked bar charts are normalized over the course of the

trajectory. The mutation of 206 from Ser to Asn dislodges the ligand from its binding site, breaking a critical H-bond with Asp231 and thus contributing to the decrease in ligand binding stability compared to that seen with the wild type enzyme. The Ser35Asn mutation that accompanies Ser206Asn is not located in the binding site, and its role in drug resistance seems to be the result of an indirect interaction between the residue and the ligand.

Fig. S5 (A) RMSF of DNDI-6174 during the 100 ns MD simulation in the mutated cytochrome *b* from Res 5 (Ser206Asn). RMSF is reported by atom number as reported in the chemical representation on the right. (B) A schematic of detailed ligand atom interactions with the protein residues. (C) RMSD plot of DNDI-6174 and cytochrome b. (D) Protein-ligand contacts plot. The possible interactions are categorized into four types: hydrogen bonds, hydrophobic, ionic and water bridges. The stacked bar charts are normalized over the course of the trajectory. See Fig. S4 legend for details of the impact of this mutation on ligand stability.

Fig. S6 (A) RMSF of DNDI-6174 during the 100 ns MD simulation in the mutated cytochrome b from Res 2 (Asp231Glu). RMSF is reported by atom number as reported in the chemical representation on the right. (B) A schematic of detailed ligand atom interactions with the protein residues. (C) RMSD plot of DNDI-6174 and cytochrome b. (D) Protein-ligand contacts plot. The possible interactions are categorized into four types: hydrogen bonds, hydrophobic, ionic and water bridges. The stacked bar charts are normalized over the course of the trajectory. Our MD analysis strongly suggests that the longer and more flexible Glu231 side chain bends away from the ligand. This new conformation of the Glu231 side chain results in a complete loss of interactions with the ligand, reflected in a 164-fold reduction in DNDI-6174 potency compared to the wild-type (Table **2**).

Fig. S7 (A) RMSF of DNDI-6174 during the 100 ns MD simulation in the mutated cytochrome *b* from Res 3 (Ser207Pro). RMSF is reported by atom number as reported in the chemical representation on the right. (B) A schematic of detailed ligand atom interactions with the protein residues. (C) RMSD plot of DNDI-6174 and cytochrome b. (D) Protein-ligand contacts plot. The possible interactions are categorized into four types: hydrogen bonds, hydrophobic, ionic and water bridges. The stacked bar charts are normalized over the course of the trajectory. The Ser207Pro mutation impacts ligand binding by changing the morphology of the binding site. The mutation causes the rearrangement of secondary structure elements, ultimately disrupting the key H-bonds interactions between the Asp231 side chain and the 2-amino group of DNDI-6174.

Fig. S8 RMSF of DNDI-6174 during the 100 ns MD simulation in the mutated cytochrome b from Res 4 (Gly31Ala). RMSF is reported by atom number as reported in the chemical representation on the right. (B) A schematic of detailed ligand atom interactions with the protein residues. (C) RMSD plot of DNDI-6174 and cytochrome b. (D) Protein-ligand contacts plot. The possible interactions are categorized into four types: hydrogen bonds, hydrophobic, ionic and water bridges. The stacked bar charts are normalized over the course of the trajectory. Our data indicates that the resistance observed in the Gly31Ala mutated results in steric clashes. The methyl group of the Ala side chain displaces the conserved water molecule bridging the interaction between the ligand and Phe34 and disrupts the interaction with Asp231 by clashes with the NH_2 of the 2-aminopyrrolopyrimidine core.

Fig. S9 Degradation profiles for DNDI-6174 incubated with human liver microsomes in the absence and presence of inhibitors specific for individual CYP isoforms. There was no significant difference in the degradation slopes ($\alpha = 0.05$) in the absence and presence of inhibitor for any isoform.

Fig. S10 Metabolite formation profiles for DNDI-6174 metabolites formed following incubation with human liver microsomes in the absence and presence of inhibitors specific for individual CYP isoforms.

Fig. S11 Experimental plasma PK data following (A) twice-daily oral administration (doses given at 6 and 24 h) for 5 days in mice and (B) once-daily oral administration for 5 days in hamsters. Note that at the highest dose of 25 (mice) and 47.3 (hamsters) mg/kg, only a single dose was administered. Symbols represent the measured data (mean ± SD, n=3) and lines represent the best-fit of the data using a one compartment body model.

Fig. S12 Plasma AUC_{24} ss and C_{max} for hamsters (qd dosing for 5 days) and mice (bid dosing at 8 and 24 h for 5 days) based on compartmental fits of the experimental data (black symbols ± SE). Experimental data on day 1 and day 5 of dosing are shown with the blue and red symbols, respectively.

Fig. S13 Simulated repeat dose plasma profiles for DNDI-6174 in mice and hamsters following once or twice-daily oral administration (8 and 24 h) for 5 days as used in the efficacy studies.

Fig. S14 Simulated human plasma concentration vs time profiles to achieve a cumulative plasma AUC of between 60 and 460 µg.h/mL. Profiles were simulated using GastroPlus and the parameters shown in Table **S16**.

Fig. S15 DNDI-6174 was tested in hIPSc-CM (cardiomyocytes) MEA (multielectrode) assay at concentrations of 0.37, 1.11, 3.33, 10 and 30 µM. Concentrations were selected based on estimated free C_{max} at efficacious exposure and potency against cardiac ion channel data: Qpatch hERG IC_{50} = 31.6 µM; Qube NaV1.5 IC_{50} > 50 µM; Qube CaV1.2 IC_{50} > 100 µM (43% inhibition). DNDI-6174 produced no notable change in the field potential duration (FPD), the spike amplitude and the beat period throughout predicting no significant QT prolongation and no notable change in cardiac conduction.

Table S1 *In vivo* efficacy of DNDI-6174 and positive controls (AmBisome or miltefosine) in (A) the acute mouse model and (B) the chronic hamster model. Data for organ burden are expressed as a % of the vehicle control in the same experiment (mean n=5 ± SEM).

Table S2 Results for the promastigote transformation assay where organs from hamsters treated with DNDI-6174, miltefosine or vehicle control were cultured *in vitro* and monitored for the emergence of viable parasites (promastigotes). Results represent an arbitrary parasite score attributed (+, ++ or +++) based on visual inspection of parasite density in the positive wells. A score of "-"is attributed in the absence of parasite. Scores are reported individually, 7 days post autopsy, from the three target organs (liver, spleen and bone-marrow).

Table S3 Summary of read counts and coverage for whole genome sequencing of DNDI-6174-resistant clones.

Table S4 Summary of non-synonymous SNPs identified in whole genome sequencing of DNDI-6174-resistant parasites.

Table S5 Physicochemical, permeability and binding properties for DNDI-6174

Table S6 *In vitro* intrinsic clearance (CLint, ± standard error of estimate) of DNDI-6174 following incubation with liver microsomes (2 independent experiments) and cryopreserved hepatocytes (single experiment) and predicted *in vivo* plasma clearance. The measured *in vivo* plasma clearance from Table **S7** is shown for reference.

Table S7 Intravenous and oral plasma pharmacokinetic properties of DNDI-6174 in mice, rats, and dogs following single dose administration.

Table S8 Oral plasma exposure of DNDI-6174 in rats following a single dose (mean n=3 ± S.D.).

Table S9 Oral plasma exposure of DNDI-6174 in dogs following a single dose (mean n=3 ± S.D.)

Table S10 Oral plasma exposure of DNDI-6174 in mice following twice daily (at 6 and 24 h) dosing for 5 days (mean n=3 ± S.D.).

Table S11 Oral plasma exposure of DNDI-6174 in hamsters following once daily dosing for 5 days (mean of n=3 ± S.D.).

Table S12 Summary of fitted plasma compartmental parameters for DNDI-6174 following single oral dosing to mice and hamsters.

Table S13 Pharmacodynamic data (liver burden, mean n=5 ± SEM) for mice infected with *L. infantum* or *L. donovani* and treated with DNDI-6174. For the liver burden data, the SEM is shown in parentheses and for the plasma PK parameters, the unbound values are shown in parentheses. Pharmacokinetic parameters are from the fitted analysis as described above.

Table S14 Pharmacodynamic data (liver burden, mean n=5 ± SEM, Table **S1**) for DNDI-6174 in hamsters infected with *L. infantum*. Pharmacokinetic parameters are from the fitted analysis as described above. Unbound PK parameters are shown in parentheses.

Table S15 Best fit parameters for the data shown in Figure 4 obtained using a 4-parameter logistic function. Values in parentheses represent the standard errors of the fitted parameters.

Table S16 Input parameters for GastroPlus simulations.

Table S17 Early *in silico* and *in vitro* cardiotoxicity assessment of DNDI-6174.

Table S18 Complete *in vitro* cytotoxicity profiling of DNDI-6174.

Table S19 DNDI-6174 profile in a panel mammalian receptors, enzymes and ion channels.

Table S20 Assessment of human complex III activity and mitochondrial toxicity.

Table S21 Cytochrome P450 inhibition by DNDI-6174.

Supplementary Materials and methods

Promastigote transformation assay

For evaluating the presence of viable residual burdens after treatment, a promastigote back-transformation assay is conducted. This test consists of the incubation at ambient temperature of aseptically collected pieces of spleen or liver tissue in 1 mL of promastigote back-transformation medium in 24-well plates. For the bone-marrow, resected femurs are flushed with 1 mL medium. The medium consists of HOMEM pH 6.0 prepared as described elsewhere (*1*) with 10% fetal bovine serum (Gibco) and 2.5% penicillin/streptomycin and 1% gentamicin (Merck). A qualitative assessment of the presence of promastigotes is made after 3 and 5 days of incubation and a score is attributed (+, ++ or +++) based on parasite density in the positive wells.

Supplementary Information on Tables

Table 1. Suppl. Info

L. infantum MHOM/FR/96/LEM3323 Cl4 was originally obtained from a French HIV-positive patient (Languedoc area in Southern France) and provided by Dr. Laurence Lachaud of the Centre National de Référence des Leishmanioses (CNRL), Montpellier, France. The miltefosine and paromomycin resistant lines (LEM3323 Cl4 MIL-R and PMM-R) were obtained by successive *in vitro* selection cycles on intracellular amastigotes (*2, 3*)

Supplementary Figures

	RES1	RES2	RES3	RES4	RES5
Ld01	1.0	1.0	1.0	1.0	1.0
Ld02	1.0	1.0	1.0	1.1	1.0
Ld03	0.5	1.0	1.0	0.5	1.0
Ld04	1.0	1.0	1.0	1.0	1.0
Ld05	1.0	1.0	1.0	1.0	1.0
Ld06	1.0	1.0	1.0	1.0	1.0
Ld07	1.0	1.0	1.0	1.0	1.0
Ld08	1.0	1.0	1.0	1.0	1.0
Ld09	1.0	1.0	1.0	1.0	1.0
Ld10	1.0	1.0	1.0	1.0	1.0
Ld11	1.0	1.0	1.0	1.0	1.0
Ld12	1.0	1.0	1.4	1.0	1.0
Ld13	1.0	1.0	1.0	1.0	1.0
Ld14	1.0	1.0	1.0	1.0	1.0
Ld15	1.0	1.0	1.0	1.0	1.0
Ld16	1.0	1.0	1.0	1.0	1.0
Ld17	1.0	1.0	1.0	1.0	1.0
Ld18	1.0	1.0	1.0	1.0	1.0
Ld19	1.0	1.0	1.0	1.0	1.0
Ld20	1.0	1.0	1.0	1.0	1.0
Ld21	1.0	1.0	1.0	1.0	1.0
Ld22	1.0	1.0	1.0	1.0	1.0
Ld23	1.0	1.0	1.0	1.0	1.0
Ld24	1.0	1.0	1.0	1.0	1.0
Ld25	1.0	1.0	1.0	1.0	1.0
Ld26	1.0	1.0	1.5	1.0	1.0
Ld27	1.0	1.0	1.0	1.0	1.0
Ld28	1.0	1.0	1.0	1.0	1.0
Ld29	1.0	1.0	1.0	1.0	1.0
Ld30	1.0	1.0	1.0	1.0	1.0
Ld31	1.0	1.0	1.0	1.0	1.0
Ld32	1.0	1.0	1.0	1.0	1.0
Ld33	1.0	1.0	1.0	1.0	1.0
Ld34	1.0	1.0	1.0	1.0	1.0
Ld35	1.0	1.0	1.0	1.0	1.0
Ld36	1.0	1.0	1.0	1.0	1.0

Fig. S1 Chromosomal CNV relative to wild-type in DNDI-6174-resistant clones.

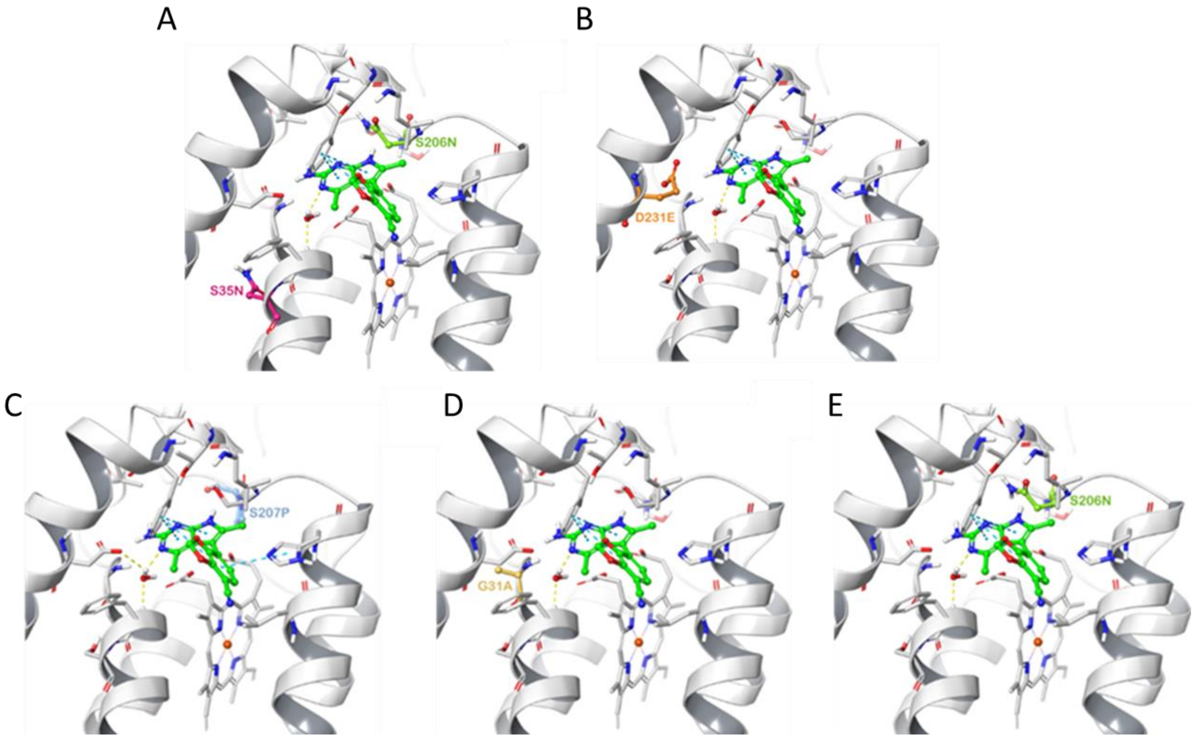

Fig. S2 Binding mode of DNDI-6174 in the Q_i sites of mutated versions of cytochrome *b* (A-E). Ser35Asn and Ser206Asn mutations identified in cell line Res 1 are reported in magenta and green. Mutation Asp231Glu identified in Res 2 is represented in orange. The Ser207Pro mutation identified in Res 3 is in light blue. Gly31Ala (Res 4) and Ser206Asn (Res 5) are reported in wheat and green, respectively.

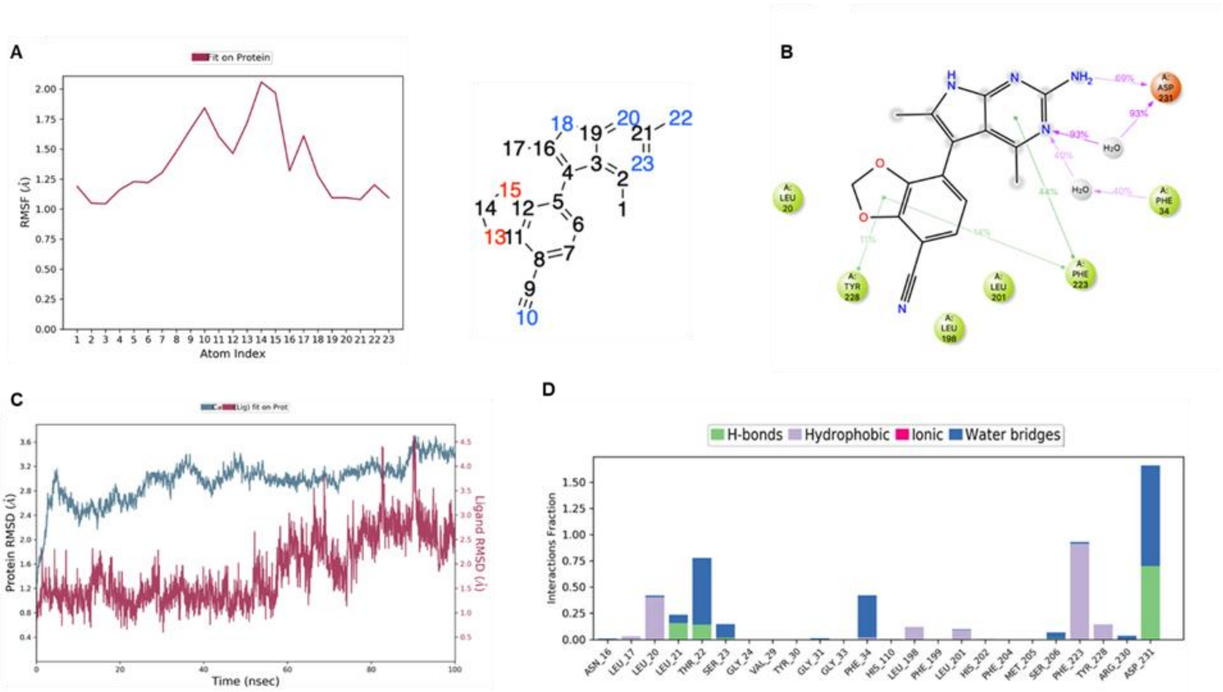

Fig. S3 (A) The Ligand Root Mean Square Fluctuation (RMSF) for each atom of DNDI-6174 in cytochrome *b* (wild-type) measuring the changes in the position of ligand atoms during the 100 ns MD simulation (atom number as reported in the chemical representation on the right). These studies indicate that benzodioxole moiety of DNDI-6174 is the portion of the molecule with the highest flexibility during binding. (B) A schematic of detailed ligand atom interactions with the amino acid residues of cytochrome *b*. (C) Root Mean Square Deviation (RMSD) plot of DNDI-6174 (right Y-axis – magenta curve) and wild-type cytochrome *b* (left Y-axis – blue curve). The plot indicates the stability of the protein and the ligand during the simulation. The overall stability of the docking binding pose is highlighted by the protein and ligand RMSD over this simulation. (D) Protein-Ligand Contacts plot. The possible interactions are categorized into four types: hydrogen bonds, hydrophobic, ionic and water bridges. The stacked bar charts are normalized over the course of the trajectory.

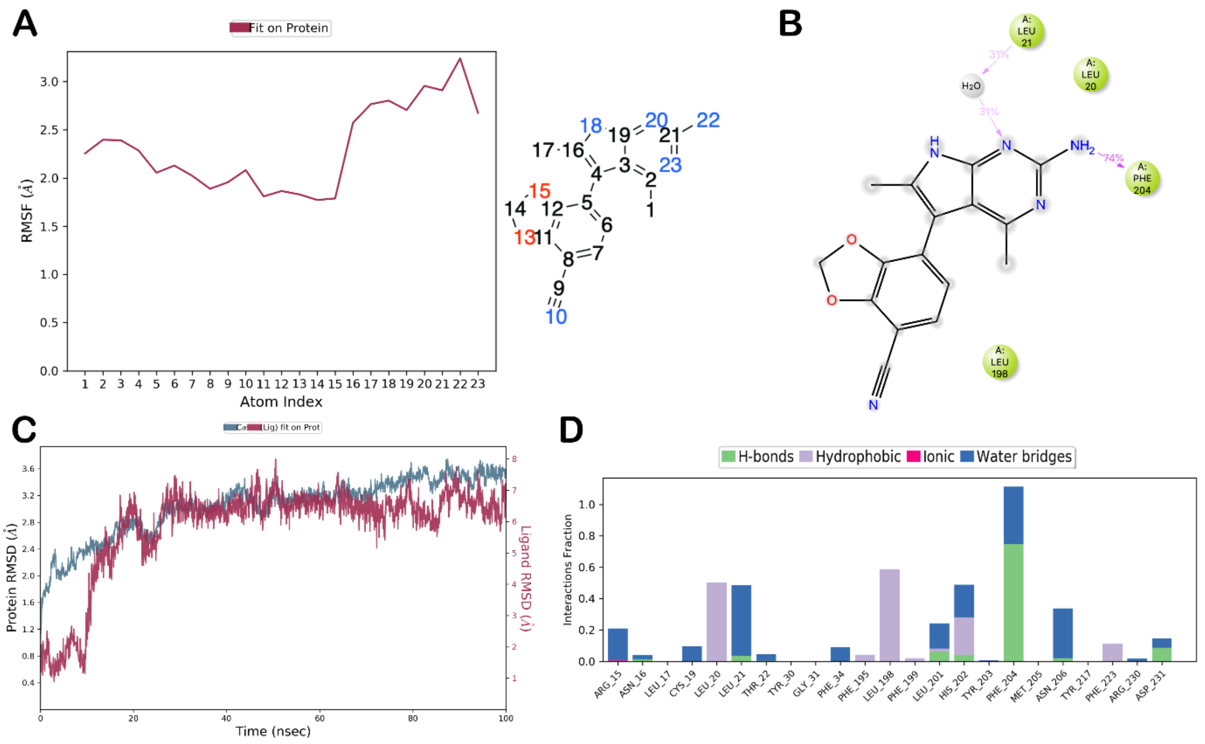

Fig. S4 (A) The Ligand Root Mean Square Fluctuation (RMSF) of DNDI-6174 during the 100 ns MD simulation in the mutated cytochrome *b* (Ser35Asn/Ser206Asn) from Res 1. Ligand flexibility in this mutated enzyme increased considerably, even for the pyrrolopyrimidine scaffold that was particularly tightly bound in the wild-type enzyme. RMSF is reported by atom number as reported in the chemical representation on the right. (B) A schematic of detailed ligand atom interactions with the protein residues. (C) Root Mean Square Deviation (RMSD) plot of DNDI-6174 and cytochrome b. (D) Protein-Ligand Contacts plot. The possible interactions are categorized into four types: hydrogen bonds, hydrophobic, ionic and water bridges. The stacked bar charts are normalized over the course of the trajectory. The mutation of 206 from Ser to Asn dislodges the ligand from its binding site, breaking a critical H-bond with Asp231 and thus contributing to the decrease in ligand binding stability compared to that seen with the wild type enzyme. The Ser35Asn mutation that accompanies Ser206Asn is not located in the binding site, and its role in drug resistance seems to be the result of an indirect interaction between the residue and the ligand.

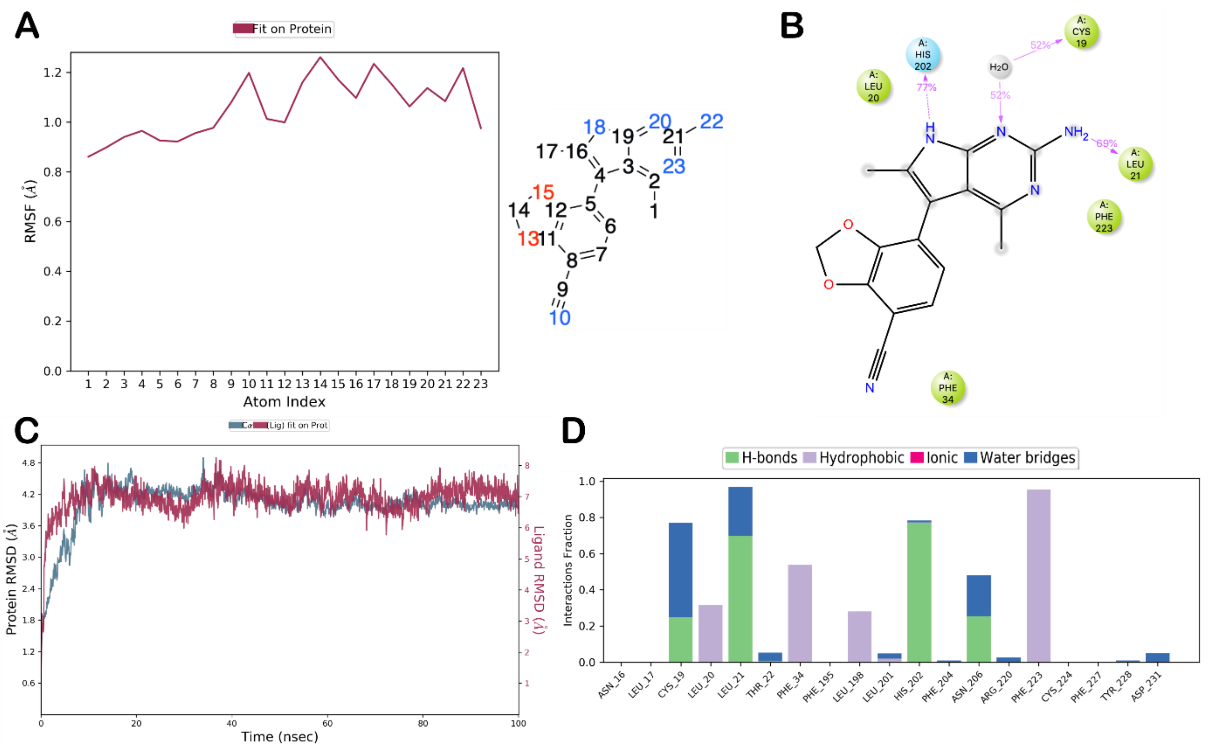

Fig. S5 (A) RMSF of DNDI-6174 during the 100 ns MD simulation in the mutated cytochrome *b* from Res 5 (Ser206Asn). RMSF is reported by atom number as reported in the chemical representation on the right. (B) A schematic of detailed ligand atom interactions with the protein residues. (C) RMSD plot of DNDI-6174 and cytochrome *b*. (D) Protein-ligand contacts plot. The possible interactions are categorized into four types: hydrogen bonds, hydrophobic, ionic and water bridges. The stacked bar charts are normalized over the course of the trajectory. See Fig. **S4** legend for details of the impact of this mutation on ligand stability.

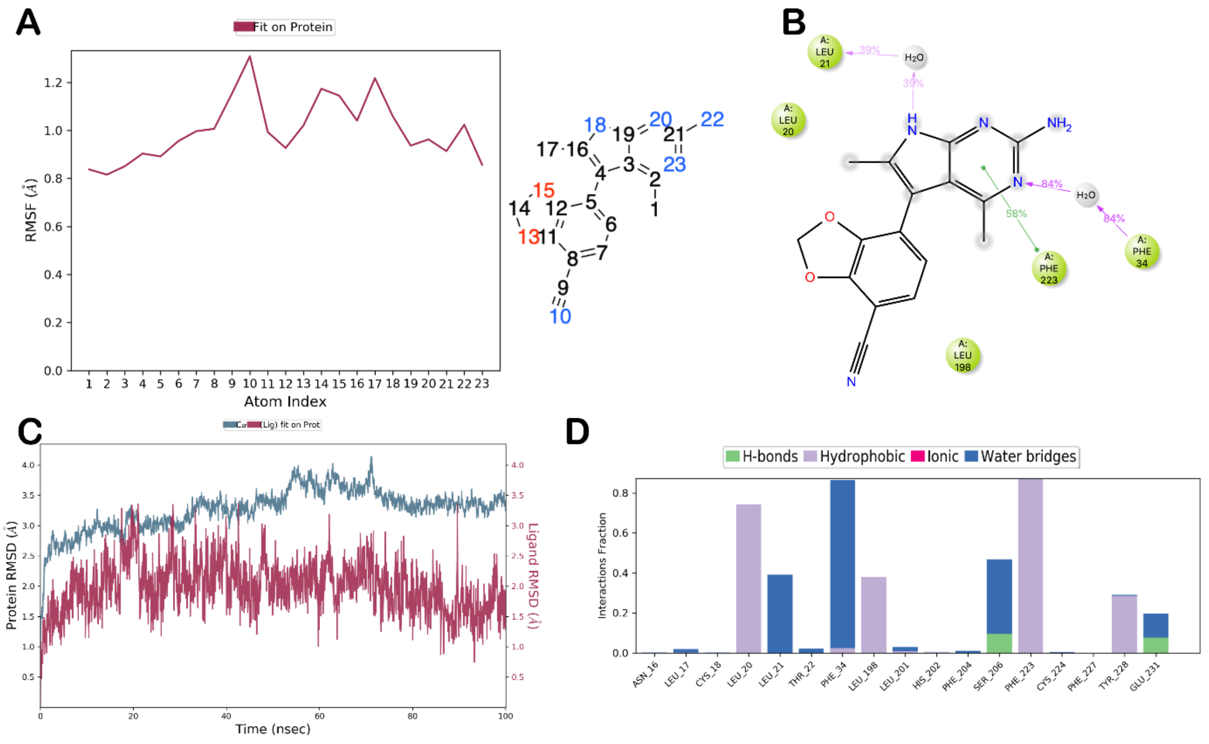

Fig. S6 (A) RMSF of DNDI-6174 during the 100 ns MD simulation in the mutated cytochrome *b* from Res 2 (Asp231Glu). RMSF is reported by atom number as reported in the chemical representation on the right. (B) A schematic of detailed ligand atom interactions with the protein residues. (C) RMSD plot of DNDI-6174 and cytochrome *b*. (D) Protein-ligand contacts plot. The possible interactions are categorized into four types: hydrogen bonds, hydrophobic, ionic and water bridges. The stacked bar charts are normalized over the course of the trajectory. Our MD analysis strongly suggests that the longer and more flexible Glu231 side chain bends away from the ligand. This new conformation of the Glu231 side chain results in a complete loss of interactions with the ligand, reflected in a 164-fold reduction in DNDI-6174 potency compared to the wild-type (Table **2**).

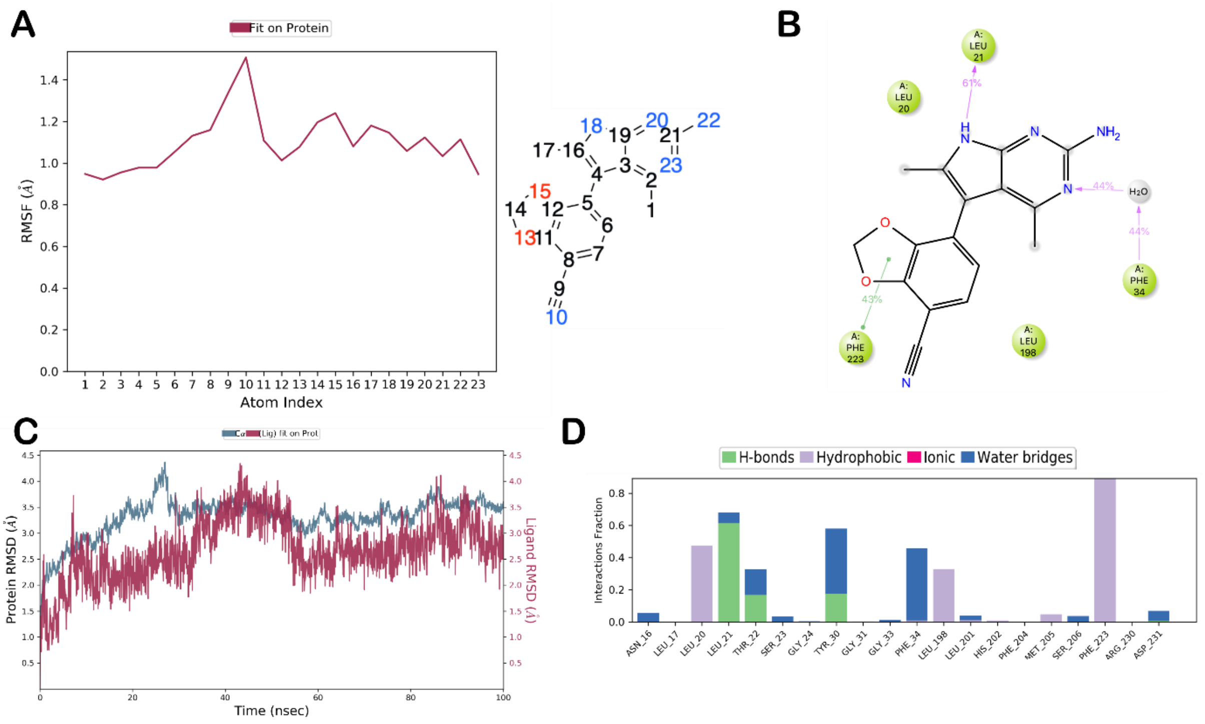

Fig. S7 (A) RMSF of DNDI-6174 during the 100 ns MD simulation in the mutated cytochrome *b* from Res 3 (Ser207Pro). RMSF is reported by atom number as reported in the chemical representation on the right. (B) A schematic of detailed ligand atom interactions with the protein residues. (C) RMSD plot of DNDI-6174 and cytochrome *b*. (D) Protein-ligand contacts plot. The possible interactions are categorized into four types: hydrogen bonds, hydrophobic, ionic and water bridges. The stacked bar charts are normalized over the course of the trajectory. The Ser207Pro mutation impacts ligand binding by changing the morphology of the binding site. The mutation causes the rearrangement of secondary structure elements, ultimately disrupting the key H-bonds interactions between the Asp231 side chain and the 2-amino group of DNDI-6174.

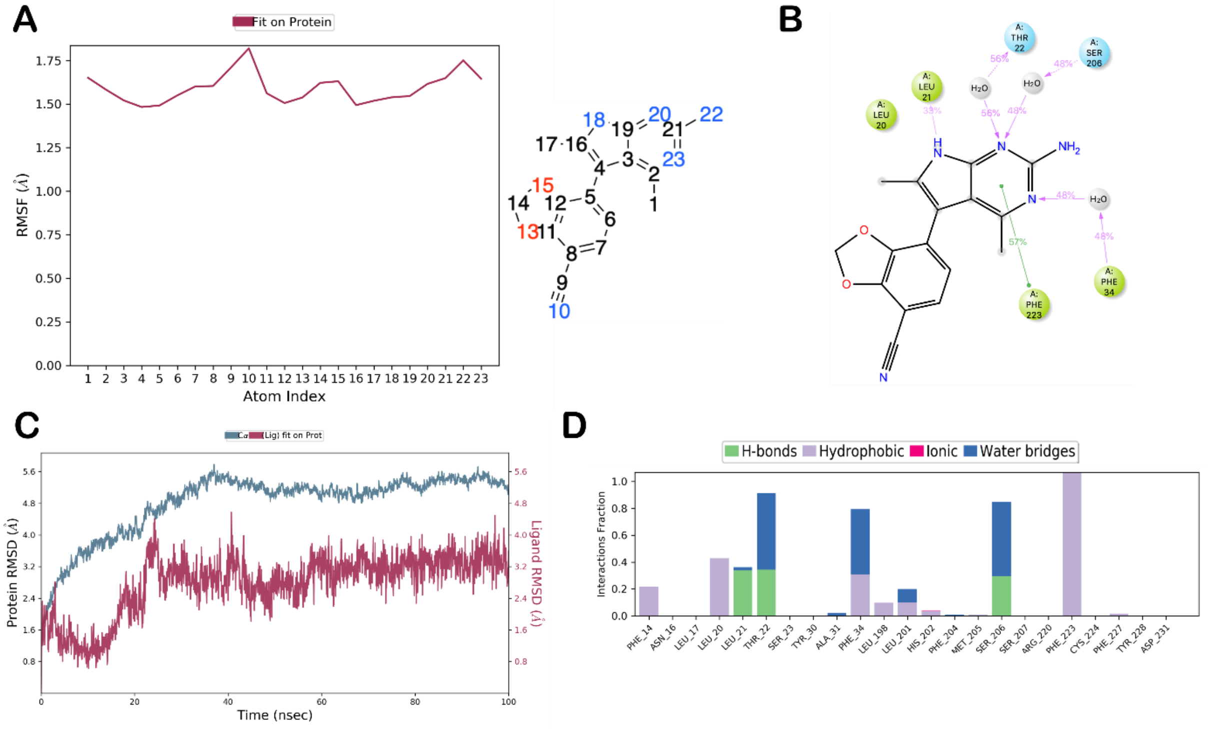

Fig. S8 RMSF of DNDI-6174 during the 100 ns MD simulation in the mutated cytochrome *b* from Res 4 (Gly31Ala). RMSF is reported by atom number as reported in the chemical representation on the right. (B) A schematic of detailed ligand atom interactions with the protein residues. (C) RMSD plot of DNDI-6174 and cytochrome *b*. (D) Protein-ligand contacts plot. The possible interactions are categorized into four types: hydrogen bonds, hydrophobic, ionic and water bridges. The stacked bar charts are normalized over the course of the trajectory. Our data indicates that the resistance observed in the Gly31Ala mutated results in steric clashes. The methyl group of the Ala side chain displaces the conserved water molecule bridging the interaction between the ligand and Phe34 and disrupts the interaction with Asp231 by clashes with the NH_2 of the 2-aminopyrrolopyrimidine core.

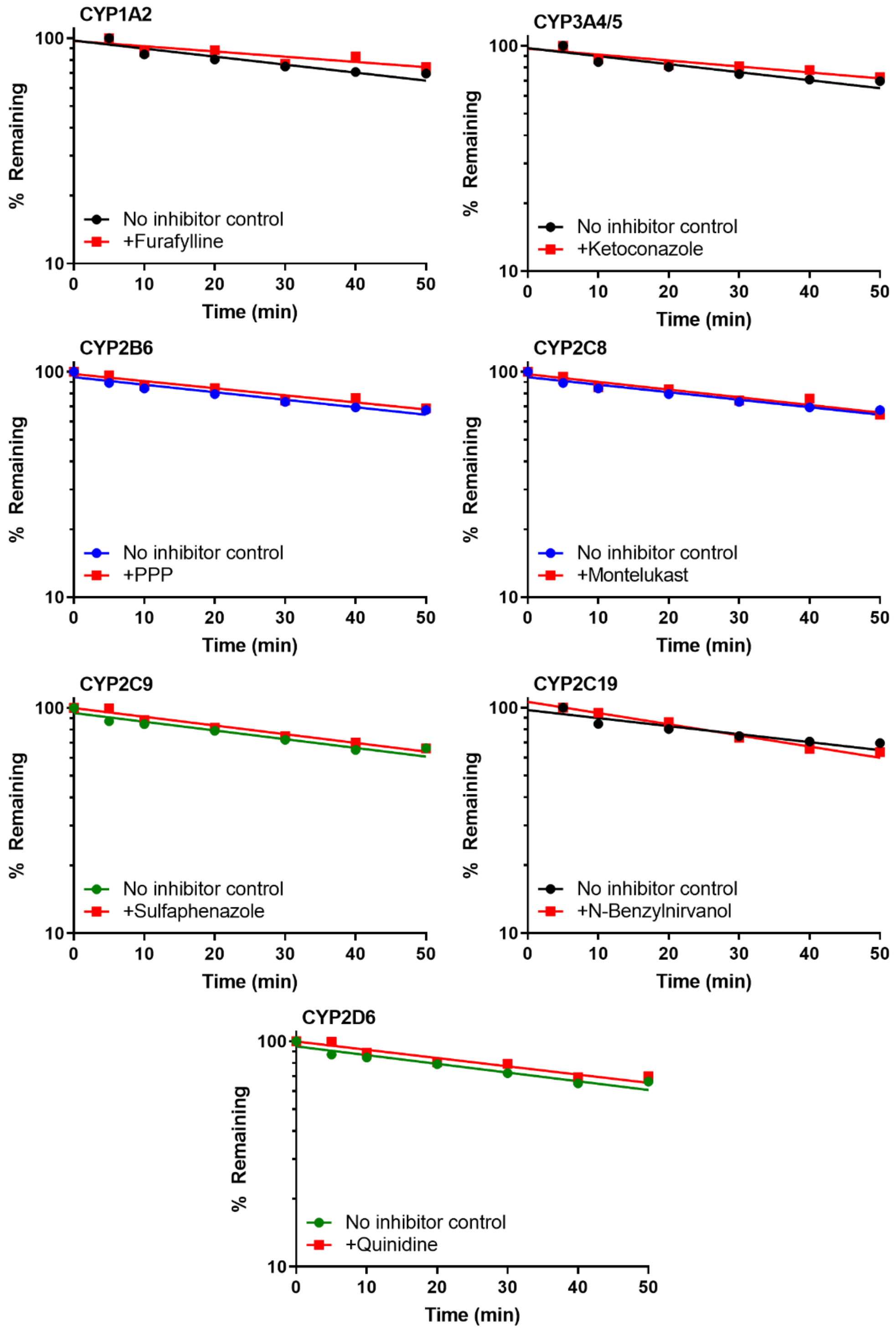

Fig. S9 Degradation profiles for DNDI-6174 incubated with human liver microsomes in the absence and presence of inhibitors specific for individual CYP isoforms. There was no significant difference in the degradation slopes ($\alpha = 0.05$) in the absence and presence of inhibitor for any isoform.

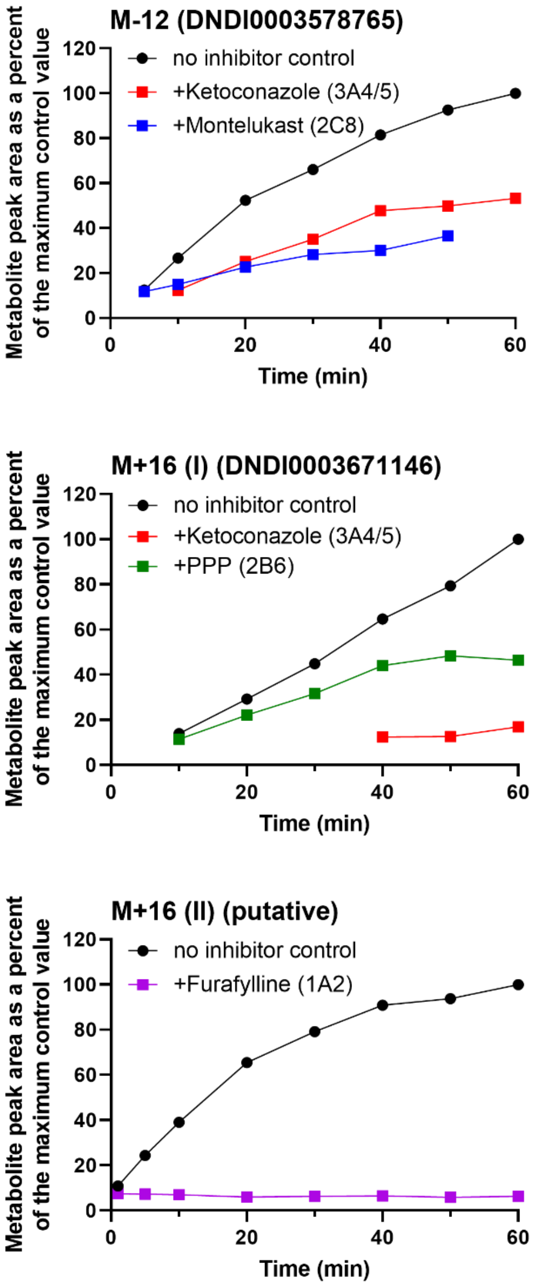

Fig. S10 Metabolite formation profiles for DNDI-6174 metabolites formed following incubation with human liver microsomes in the absence and presence of inhibitors specific for individual CYP isoforms.

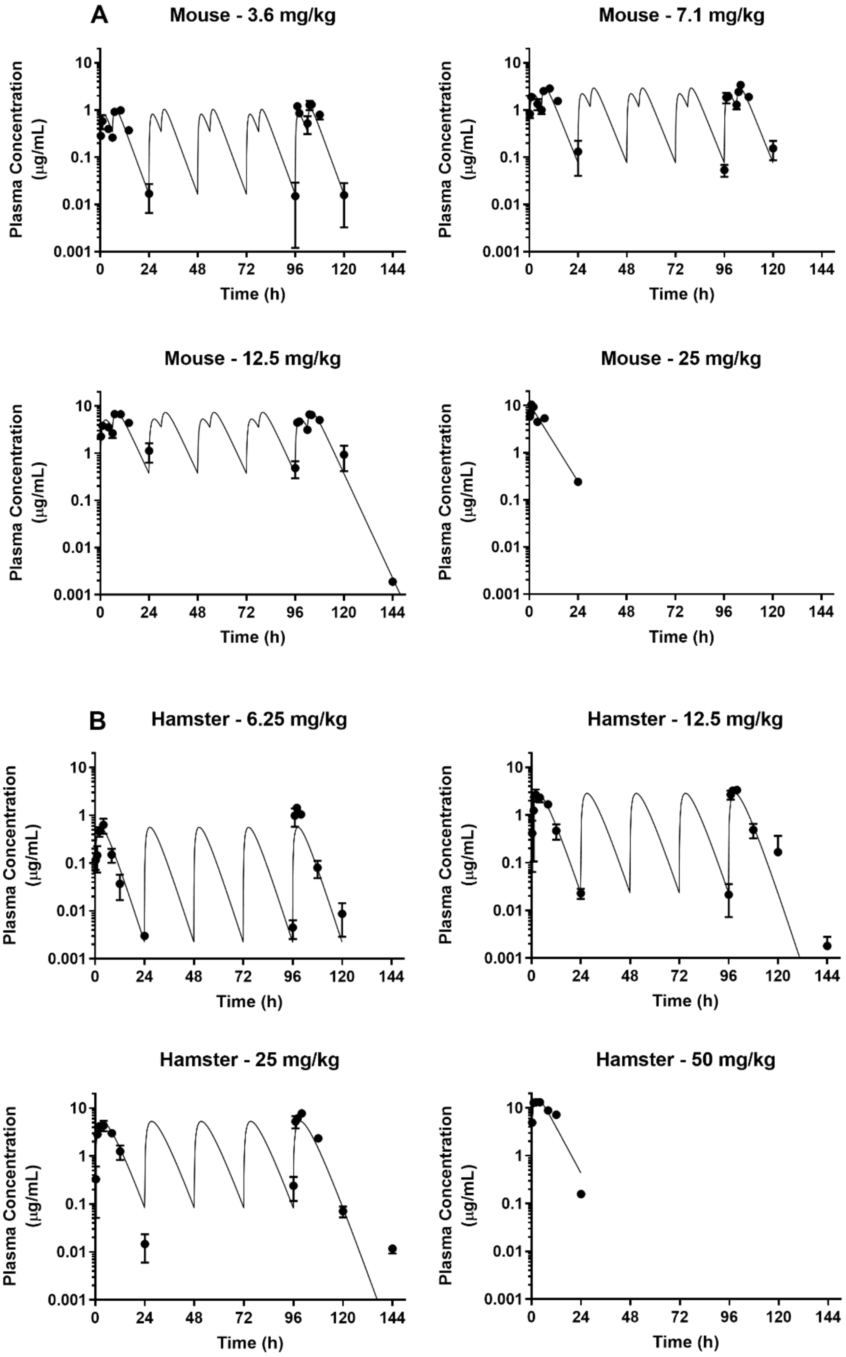

Fig. S11 Experimental plasma PK data following (A) twice-daily oral administration (doses given at 6 and 24 h) for 5 days in mice and (B) once-daily oral administration for 5 days in hamsters. Note that at the highest dose of 25 (mice) and 47.3 (hamsters) mg/kg, only a single dose was administered. Symbols represent the measured data (mean ± SD, n=3) and lines represent the best-fit of the data using a one compartment body model.

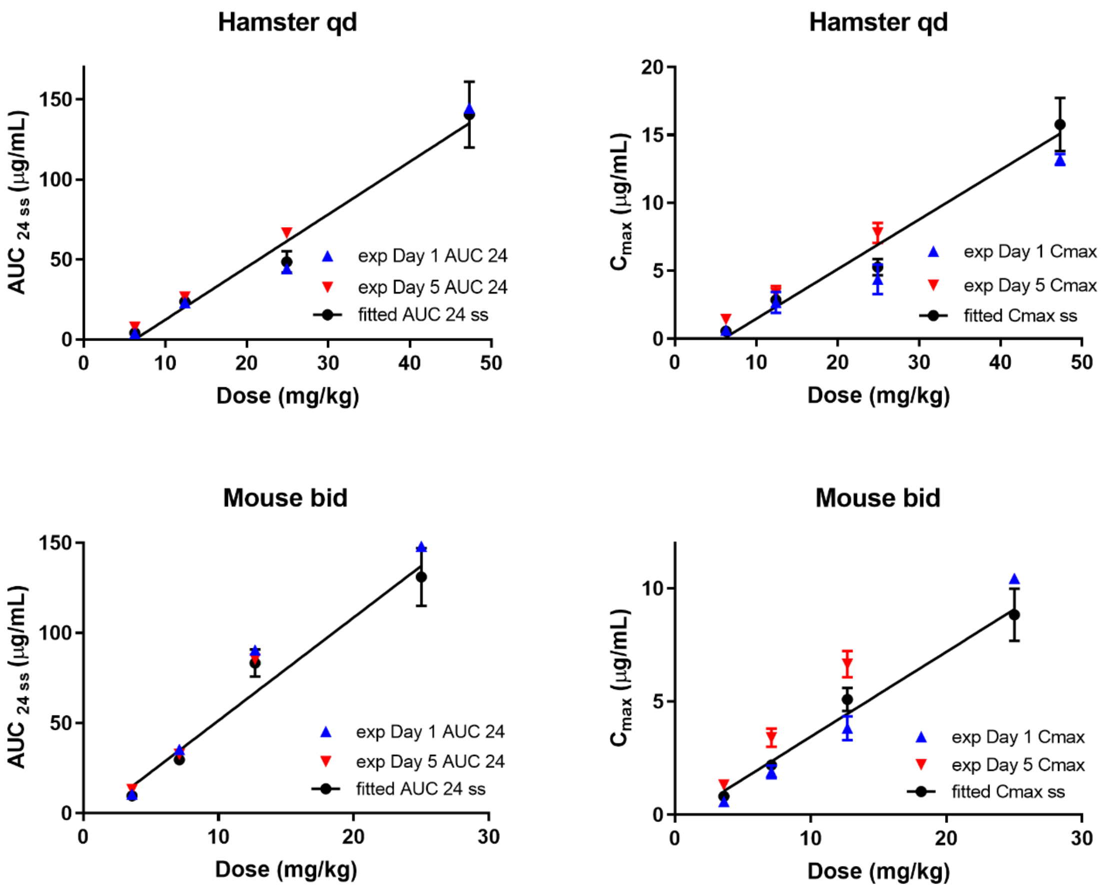

Fig. S12 Plasma AUC_{24} ss and C_{max} for hamsters (qd dosing for 5 days) and mice (bid dosing at 8 and 24 h for 5 days) based on compartmental fits of the experimental data (black symbols ± SE). Experimental data on day 1 and day 5 of dosing are shown with the blue and red symbols, respectively.

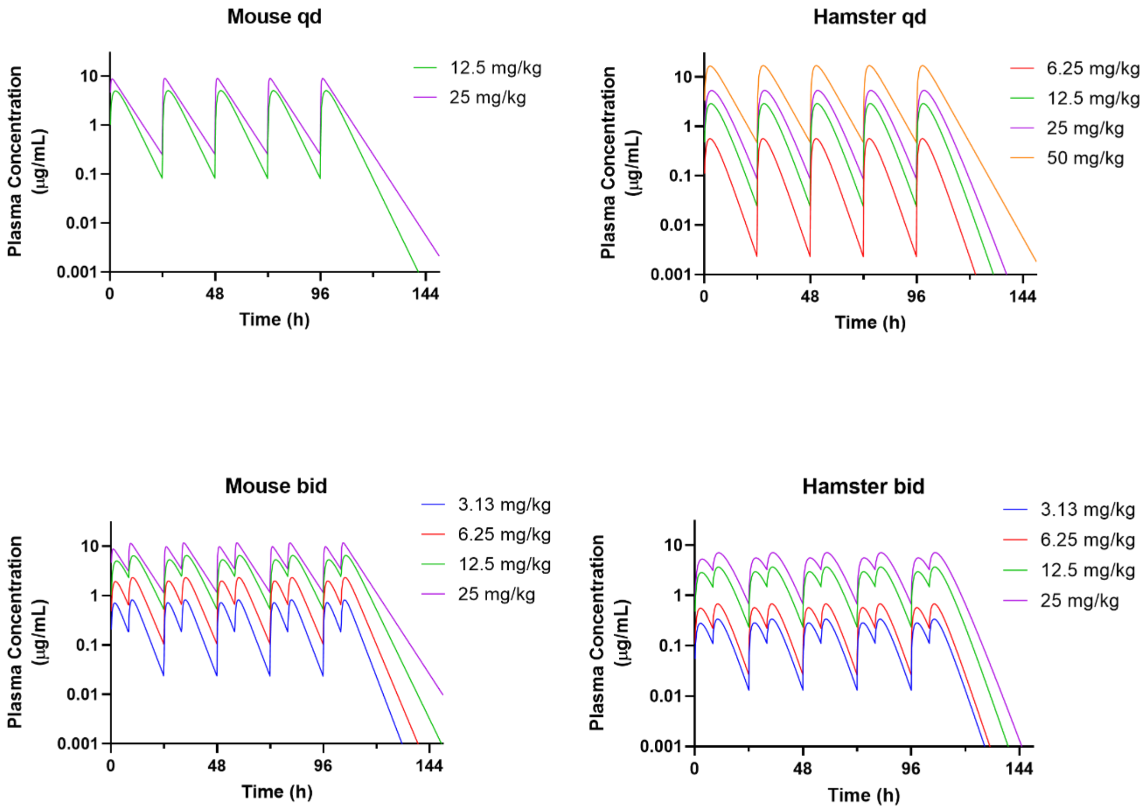

Fig. S13 Simulated repeat dose plasma profiles for DNDI-6174 in mice and hamsters following once or twice-daily oral administration (8 and 24 h) for 5 days as used in the efficacy studies.

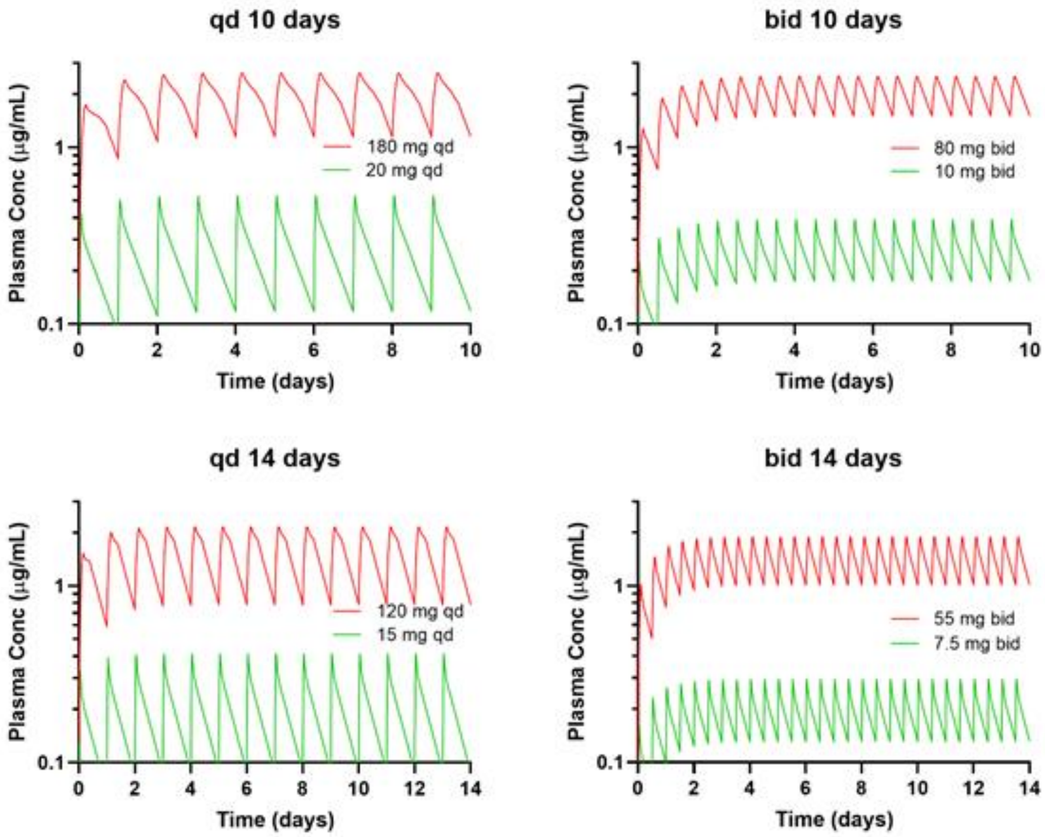

Fig. S14 Simulated human plasma concentration vs time profiles to achieve a cumulative plasma AUC of between 60 and 460 µg.h/mL. Profiles were simulated using GastroPlus and the parameters shown in Table **S16**.

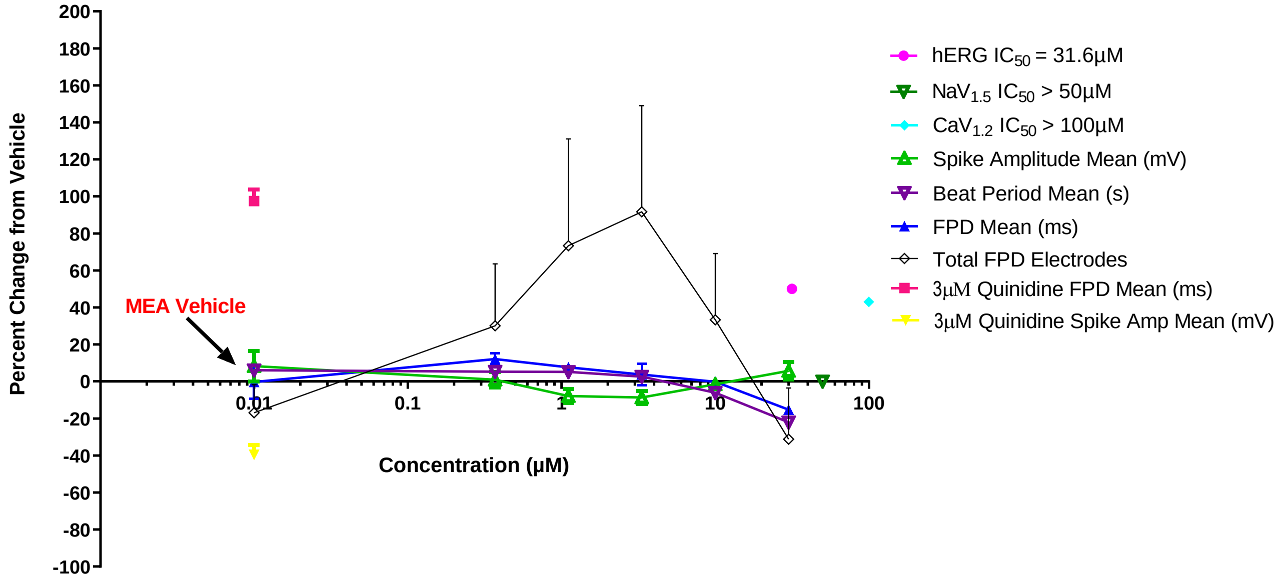

Fig. S15 DNDI-6174 was tested in the hIPSc-CM (cardiomyocytes) MEA (multielectrode) assay at concentrations of 0.37, 1.11, 3.33, 10 and 30 µM. Concentrations were selected based on the estimated free C_{max} at the efficacious exposure and potency against cardiac ion channel data: Qpatch hERG IC_{50} = 31.6 µM; Qube NaV1.5 IC_{50} > 50 µM; Qube CaV1.2 IC_{50} > 100 µM (43% inhibition). DNDI-6174 produced no notable change in the field potential duration (FPD), the spike amplitude and the beat period throughout, predicting no significant QT prolongation and no notable change in cardiac conduction.

Supplementary Tables

Table S1 *In vivo* efficacy of DNDI-6174 and positive controls (AmBisome or miltefosine) in (A) the acute mouse model and (B) the chronic hamster model. Data for organ burden are expressed as a % of the vehicle control in the same experiment (mean n=5 ± SEM).

A. Acute mouse model

Species	Compound	Dose (mg/kg)	Regimen	Duration	Liver Burden % of Vehicle Control (SEM)
L. infantum	miltefosine	40	qd	5	1.42 (0.46)
L. infantum	miltefosine	20	qd	10	0.71 (0.25)
L. infantum	DNDI-6174	25	bid	5	0.60 (0.28)
L. infantum	DNDI-6174	12.5	bid	5	1.07 (0.23)
L. infantum	DNDI-6174	6.25	bid	5	15.1 (1.19)
L. infantum	DNDI-6174	25	qd	5	0.96 (0.28)
L. infantum	DNDI-6174	12.5	qd	5	11.80 (2.22)
L. infantum	DNDI-6174	6.25	bid	10	2.59 (0.47)
L. infantum	DNDI-6174	3.125	bid	10	41.9 (6.25)
L. donovani	AmBisome	1	qd	5	1.98 (0.622)
L. donovani	AmBisome	10	qd	5	14.7 (2.99)
L. donovani	DNDI-6174	25	bid	5	0.16 (0.11)
L. donovani	DNDI-6174	12.5	bid	5	0.40 (0.34)
L. donovani	DNDI-6174	6.25	bid	5	45.50 (5.33)

B. Chronic hamster model

Species	Compound	Dose (mg/kg)	Regimen	Duration	Liver Burden % of Vehicle (SEM)	Spleen Burden % of Vehicle (SEM)	Bone-marrow Burden % of Vehicle (SEM)
L. infantum	miltefosine	40	qd	5	2.22 (0.78)	1.18 (0.44)	5.54 (1.85)
L. infantum	DNDI-6174	50	qd	5	0.28 (0.09)	0.15 (0.04)	0.11 (0.06)
L. infantum	DNDI-6174	25	qd	5	0.36 (0.17)	0.05 (0.03)	0.63 (0.34)
L. infantum	DNDI-6174	12.5	bid	5	0.34 (0.10)	0.13 (0.06)	0.37 (0.13)
L. infantum	DNDI-6174	12.5	qd	5	1.87 (0.53) *	0.52 (0.08)	1.03 (0.35) *
L. infantum	DNDI-6174	6.25	bid	5	3.13 (1.17) *	2.37 (1.33)	6.38 (3.56)
L. infantum	DNDI-6174	6.25	qd	5	33.60 (9.11)	34.90 (9.46)	42.00 (12.90)
L. infantum	DNDI-6174	3.125	bid	5	78.30 (19.90)	61.00 (12.50)	69.00 (10.40)

*n=4 for each dose (outliers based on Grubbs' test excluded from means and SEM)

Table S2 Results for the promastigote back-transformation assay where organs from hamsters treated with DNDI-6174, miltefosine or vehicle control were cultured *in vitro* and monitored for the emergence of viable promastigotes. Results represent an arbitrary parasite score attributed (+, ++ or +++) based on visual inspection of parasite density in the positive wells. A score of "-"is attributed in the absence of parasite. Scores are reported individually, 7 days post autopsy, from the three target organs (liver, spleen and bone-marrow).

Oral treatment given for 5 days	Individual ID	Liver	Spleen	Bone-marrow
Vehicle (negative control)	1	++	+	++
	2	+	+	++
	3	+	+	+++
	4	++	+++	++
	5	++	+++	+++
	6	+	++	++
	7	++	++	++
	8	+	++	++
	9	-	++	++
	10	+	++	++
Miltefosine 40 mg/kg, qd (positive control)	1	+	+	+
	2	+	-	+
	3	-	+	-
	4	-	+	++
	5	-	+	+
	6	-	++	++
	7	+	++	-
	8	-	++	++
	9	-	++	++
	10	-	+	+
DNDI-6174 50 mg/kg, qd	1	-	-	-
	2	-	+	+
	3	-	-	-
	4	+	-	-
	5	-	-	-
DNDI-6174 25 mg/kg, qd	1	-	-	+
	2	+	-	-
	3	-	-	-
	4	-	-	-
	5	-	-	-
DNDI-6174 12.5 mg/kg, bid	1	-	-	-
	2	-	-	-

	3	-	+	-
	4	-	-	-
	5	-	-	+
DNDI-6174 12.5 mg/kg, qd	1	+	++	+
	2	-	+	+
	3	+	++	++
	4	+	++	++
	5	-	++	+
DNDI-6174 6.25 mg/kg, bid	1	+	++	++
	2	-	-	-
	3	-	-	-
	4	+	++	++
DNDI-6174 6.25 mg/kg, qd	1	+	++	++
	2	+	+	++
	3	+	++	++
	4	++	++	++
	5	+	++	++
DNDI-6174 3.125 mg/kg, bid	1	+	++	++
	2	+	++	++
	3	+	++	++
	4	-	++	++
	5	-	++	++

Table S3 Summary of read counts and coverage for whole genome sequencing of DNDI-6174-resistant clones.

Cell line	Number of reads	Read length	Percentage mapped (%)	Fold coverage	Gain of SNP homozygosity	Gain of SNP heterozygosity
WT	40060512	100	85.58	106	-	-
Res 1	41088566	100	85.07	108	2	1
Res 2	40712918	100	85.22	107	1	0
Res 3	41218758	100	86.82	110	1	1
Res 4	41261880	100	85.35	108	1	2
Res 5	40778210	100	84.09	106	1	1

Table S4 Summary of non-synonymous SNPs identified in whole genome sequencing of DNDI-6174-resistant parasites

Chromosome	Chromosome position	Reference	Mutation	Amino acid change	Res 1	Res 2	Res 3	Res 4	Res 5	Gene ID	Gene name
Kinetoplast	9121	G	C	Gly31Ala	0/0	0/0	0/0	1/1	0/0	-	Cytochrome *b*
Kinetoplast	9133	G	A	Ser35Asn	1/1	0/0	0/0	0/0	0/0	-	Cytochrome *b*
Kinetoplast	9646	G	A	Ser206Asn	1/1	0/0	0/0	0/0	1/1	-	Cytochrome *b*
Kinetoplast	9648	T	C	Ser207Pro	0/0	0/0	1/1	0/0	0/0	-	Cytochrome *b*
Kinetoplast	9722	T	A	Asp231Glu	0/0	1/1	0/0	0/0	0/0	-	Cytochrome *b*
Ld09_v01s1	432417	C	T	Arg277His	0/1	0/0	0/0	0/0	0/0	LdBPK_091080.1	LEM3 (ligand-effect modulator 3) family / CDC50 family
Ld20_v01s1	649823	T	G	Val392Gly	0/1	0/0	0/0	0/0	0/1	LdBPK_201460.1	hypothetical protein
Ld32_v01s1	403316	G	A	Cys586Tyr	0/0	0/0	0/0	0/1	0/0	LdBPK_321050.1	WD domain, G-beta repeat
Ld34_v01s1	1501530	A	G	Tyr273Cys	0/0	0/0	0/1	0/0	0/0	LdBPK_343690.1	Dynein heavy chain
Ld35_v01s1	1748758	G	A	Ala104Thr	0/0	0/0	0/0	0/1	0/0	LdBPK_354400.1	pre-RNA processing PIH1/Nop17

Table S5 Physicochemical, permeability and binding properties for DNDI-6174

Parameter	Value	Parameter	Value
Molecular weight (Da)	307.3	Plasma fraction unbound	
H-bond donors/acceptors	2 / 6	Human	0.059
Polar surface area (Å^2)	111	Dog	0.050
Calc Log P	1.8	Hamster	0.090
Log $D_{7.4}$ (calc / measured)	1.5 / 2.6	Rat	0.069
pKa (calc / measured)	7.5 / 5.6	Mouse	0.120
Solubility		Caco-2 permeability	
FaSSGF (pH 1.6) (mg/mL)	1.2	A-B P_{app} (cm/s)	71
FaSSIF (pH 6.5) (mg/mL)	0.011	A-B Mass balance (%)	98
FeSSIF (pH 5.8) (mg/mL)	0.054	B-A P_{app} (cm/s)	72
PBS (pH 7.4) (mg/mL)	0.0065	B-A Mass balance (%)	96
		Efflux ratio	1.0
Blood to Plasma Ratio		Microsome/hepatocyte fraction unbound	0.51 / 0.63
Human	0.80		
Dog	0.87		
Hamster	NA		
Rat	0.93		
Mouse	0.88		

Table S6 *In vitro* intrinsic clearance (CL_{int}, ± standard error of estimate) of DNDI-6174 following incubation with liver microsomes (2 independent experiments) and cryopreserved hepatocytes (single experiment) and predicted *in vivo* plasma clearance. The measured *in vivo* plasma clearance from Table **S7** is shown for reference.

Species	*In vitro* Microsome CL_{int} (µL/min/mg)	Microsome predicted *in vivo* CL_{plasma} (mL/min/kg)	*In vitro* Hepatocyte CL_{int} (µL/min/10^6 cells)	Hepatocyte predicted *in vivo* CL_{plasma} (mL/min/kg)	Measured *in vivo* CL_{plasma} (mL/min/kg)
Human	6.4 ± 0.75<br>7.2 ± 1.2	0.86	1.1 ± 0.27	0.31	---
Dog	8.3 ± 0.65<br>4.8 ± 0.47	0.91	1.0 ± 0.27	0.30	3.4
Rat	10 ± 0.79<br>10 ± 0.37	2.4	5.3 ± 0.19	2.7	2.5
Mouse	17 ± 0.59<br>13 ± 0.38	12	9.7 ± 0.091	17	7.5

Table S7 Intravenous and oral plasma pharmacokinetic properties of DNDI-6174 in mice, rats, and dogs following single dose administration

Intravenous						
Parameter	Mouse [a,b]		Rat [b]		Dog [c]	
	Mean	S.D.	Mean	S.D.	Mean	Individual
Dose (mg/kg)	2.0	---	3.0	---	1.0	---
Plasma CL (mL/min/kg)	7.46	---	2.12 [b]<br>2.95 [c]	0.18<br>0.15	3.5	3.57, 3.13
Plasma Vss (L/kg)	1.12	---	1.40 [b]<br>1.30 [c]	0.08<br>0.11	2.50	2.02, 2.99
Apparent $t_{1/2}$ (h)	2.0	---	7.9 [b]<br>5.3 [c]	0.21<br>0.36	9.84	6.48, 13.2
Plasma AUC_{0-inf} (µg.h/mL)	4.47	---	23.6 [b]<br>18.5 [c]	1.95<br>0.947	5.00	4.67, 5.33

Oral							
Parameter	Mouse [a,b]	Rat [c]		Dog [c]		Hamster [c]	
		Mean	S.D.	Mean	Individual	Mean	S.D.
Dose (mg/kg)	25	10	---	5.0	---	12.5	---
C_{max} (µg/mL)	10.4	4.21	0.284	2.02	1.20, 2.83	2.87	0.425
T_{max} (h)	1.0	4.0	0	4.0	4.0, 4.0	2.7	1.2
Apparent $t_{1/2}$ (h)	3.7	7.0	0.78	6.9	6.6, 7.3	2.6	0.17
Plasma $AUC_{0-\infty}$ (µg.h/mL)	75.5	57.7	9.39	18.5	10.9, 26.0	21.2	3.97
Bioavailability (%)	>100	93	15	90	52, 128	---	---

[a] Sparse sampling with n=2 mice per time point; data are based on the mean data
[b] Monash University; nominal dose (n=3 rats)
[c] WuXi AppTec; nominal dose (n=3 rats, n=2 dogs, n=3 hamsters)

Table S8 Oral plasma exposure of DNDI-6174 in rats following a single dose (mean n=3 ± S.D.)

Parameter	50 mg/kg [a]		100 mg/kg [a]		300 mg/kg [a]	
	Mean	S.D.	Mean	S.D.	Mean	S.D.
C_{max} (µg/mL)	14.2	2.95	20.9	1.60	30.9	4.81
T_{max} (h)	5.7	4.0	17	12	27	20
$T_{1/2}$ (h)	9.05	1.29	c.n.d.	---	c.n.d.	---
$AUC_{0-\infty}$ (µg.h/mL)	292	43.8	430 [b]	22.5	562 [b]	88.1

[a] WuXi AppTec; nominal dose
[b] the terminal phase was not well-defined; AUC_{0-24} is reported
c.n.d. = could not determine due to insufficient definition of the terminal phase

Table S9 Oral plasma exposure of DNDI-6174 in dogs following a single dose (mean n=3 ± S.D.)

Parameter	30 mg/kg [a]		90 mg/kg [a]	
	Mean	S.D.	Mean	S.D.
C_{max} (µg/mL)	9.59	2.76	19.1	5.88
T_{max} (h)	3.33	1.15	10.7	11.5
$T_{1/2}$ (h)	14.4	2.8	14.2, 20.9 [b]	---
$AUC_{0-\infty}$ (µg.h/mL)	255	95.1	613, 533 [b]	---

[a] WuXi AppTec; nominal dose
[b] the terminal phase was not well defined in the third dog

Table S10 Oral plasma exposure of DNDI-6174 in mice following twice daily (at 6 and 24 h) dosing for 5 days (mean n=3 ± S.D.)

Parameter	3.6 mg/kg bid [a]		7.1 mg/kg bid [a]		12.5 bid mg/kg [b]	
	Mean	S.D.	Mean	S.D.	Mean	S.D.
C_{max} (first dose) (µg/mL)	0.600	0.167	1.93	0.238	3.83	0.508
C_{max} (last dose) (µg/mL)	1.35	0.182	3.04	0.409	6.81	0.456
$AUC_{0\text{-}24}$ (first dose) (µg.h/mL)	10.6	0.899	35.5	2.31	94.5	6.93
$AUC_{96\text{-}120}$ (last dose) (µg.h/mL)	15.7	2.37	37.0	2.44	93.3	9.64

[a] WuXi AppTec; average measured dose determined on days 1, 4, and 5
[b] WuXi AppTec; nominal dose

Table S11 Oral plasma exposure of DNDI-6174 in hamsters following once daily dosing for 5 days (mean of n=3 ± S.D.)

Parameter	6.25 qd mg/kg [a]		12.5 qd mg/kg [a]		25 qd mg/kg [a]		50 mg/kg [a,b,c]	
	Mean	S.D.	Mean	S.D.	Mean	S.D.	Mean	S.D.
C_{max} (Day 1) (µg/mL)	0.661	0.194	2.87	0.425	4.56	1.03	13.2	0.400
C_{max} (Day 5) (µg/mL)	1.43	0.181	3.53	0.208	7.79	0.735	---	---
$AUC_{0\text{-}24}$ (first dose) (µg.h/mL)	3.58	0.334	22.9	4.37	44.2	3.61	145	---
$AUC_{96\text{-}120}$ (last dose) (µg.h/mL)	9.23	0.797	30.2	5.02	77.4	3.35	---	---

[a] WuXi AppTec; nominal dose
[b] Single dose on day 1 only
[c] Sparse sampling with n=3 samples per time point

Table S12 Summary of fitted plasma compartmental parameters for DNDI-6174 following single oral dosing to mice and hamsters.

		Mice		
Parameter	3.6 mg/kg	7.1 mg/kg	12.7 mg/kg	25 mg/kg
K01 (h^{-1})	1.04	0.833	0.634	3.00
K10 (h^{-1})	0.270	0.246	0.213	0.158
V/F (L/kg)	2.76	1.95	1.43	2.40
CL/F (mL/h/kg)	744	479	305	381
AUC_{∞} (µg.h/mL)	4.84	14.8	41.7	65.7
C_{max} (µg/mL)	0.813	2.19	5.10	8.83
		Hamsters		
Parameter	6.25 mg/kg	12.5 mg/kg	25 mg/kg	50 mg/kg
K01 (h^{-1})	0.304	0.321	0.298	0.660
K10 (h^{-1})	0.481	0.336	0.295	0.184
V/F (L/kg)	3.20	1.57	1.75	1.83
CL/F (mL/h/kg)	1538	525	515	337
AUC_{∞} (µg.h/mL)	4.07	23.6	48.4	140
C_{max} (µg/mL)	0.563	2.85	5.28	15.6

Table S13 Pharmacodynamic data (liver burden, mean n=5 ± SEM) for mice infected with *L. infantum* or *L. donovani* and treated with DNDI-6174. For the liver burden data, the SEM is shown in parentheses and for the plasma PK parameters, the unbound values are shown in parentheses. Pharmacokinetic parameters are from the fitted analysis as described above.

Strain	Dose (mg/kg)	Regimen	Duration	Liver Burden as a % of Vehicle Control (SEM)	C_{max} (µg/mL) (unbound)	C_{min} (µg/mL) (unbound)	$C_{av\ ss}$ (µg/mL) (unbound)	$AUC_{24\ ss}$ (µg.h/mL) (unbound)	Cum AUC (µg.h/mL) (unbound)
L. infantum	25	bid	5	0.595 (0.282)	11.7 (1.41)	1.14 (0.137)	5.47 (0.657)	131 (15.8)	657 (78.8)
L. infantum	12.5	bid	5	1.07 (0.226)	6.50 (0.780)	0.520 (0.062)	3.42 (0.410)	82.0 (9.84)	410 (49.2)
L. infantum	6.25	bid	5	15.1 (1.19)	2.33 (0.279)	0.102 (0.012)	1.09 (0.130)	26.1 (3.13)	131 (15.7)
L. infantum	25	qd	5	0.955 (0.279)	9.03 (1.08)	0.251 (0.030)	2.74 (0.328)	65.7 (7.88)	328 (39.4)
L. infantum	12.5	qd	5	11.8 (2.22)	5.07 (0.608)	0.080 (0.010)	1.71 (0.205)	41.0 (4.92)	205 (24.6)
L. infantum	6.25	bid	10	2.59 (0.47)	2.33 (0.279)	0.102 (0.012)	1.09 (0.130)	26.1 (3.13)	261 (31.3)
L. infantum	3.125	bid	10	41.9 (6.25)	0.820 (0.098)	0.023 (0.003)	0.351 (0.042)	8.41 (1.01)	84.1 (10.1)
L. donovani	25	bid	5	0.164 (0.107)	11.7 (1.41)	1.14 (0.137)	5.47 (0.657)	131 (15.8)	657 (78.8)
L. donovani	12.5	bid	5	0.403 (0.339)	6.50 (0.780)	0.520 (0.062)	3.42 (0.410)	82.0 (9.84)	410 (49.2)
L. donovani	6.25	bid	5	45.5 (5.33)	2.33 (0.279)	0.102 (0.012)	1.09 (0.130)	26.1 (3.13)	131 (15.7)

Table S14 Pharmacodynamic data (liver burden, mean n=5 ± SEM (Table S2)) for DNDI-6174 in hamsters infected with *L. infantum*. Pharmacokinetic parameters are from the fitted analysis as described above. Unbound PK parameters are shown in parentheses.

Strain	Dose (mg/kg)	Regimen	Duration	Liver Burden as a % of Vehicle Control (SEM)	C_{max} (µg/mL) (unbound)	C_{min} (µg/mL) (unbound)	$C_{av\ ss}$ (µg/mL) (unbound)	$AUC_{24\ ss}$ (µg.h/mL) (unbound)	Cum AUC (µg.h/mL) (unbound)
L. infantum	50	qd	5	0.280 (0.089)	17.0 (1.53)	0.463 (0.042)	6.19 (0.557)	148 (13.4)	742 (66.8)
L. infantum	25	qd	5	0.364 (0.169)	5.33 (0.480)	0.083 (0.007)	2.02 (0.182)	48.6 (4.37)	243 (21.9)
L. infantum	12.5	bid	5	0.343 (0.098)	3.69 (0.332)	0.238 (0.021)	1.98 (0.178)	47.6 (4.28)	238 (21.4)
L. infantum	12.5	qd	5	1.87 (0.529) *	2.88 (0.259)	0.023 (0.002)	0.991 (0.089)	23.8 (2.14)	119 (10.7)
L. infantum	6.25	bid	5	3.13 (1.17) *	0.682 (0.061)	0.027 (0.002)	0.339 (0.030)	8.13 (0.731)	40.6 (3.66)
L. infantum	6.25	qd	5	33.6 (9.11)	0.563 (0.051)	0.0022 (0.0002)	0.169 (0.015)	4.06 (0.366)	20.3 (1.83)
L. infantum	3.125	bid	5	78.3 (19.9)	0.341 (0.031)	0.013 (0.001)	0.169 (0.015)	4.06 (0.366)	20.3 (1.83)

*n=4 for each dose (outliers based on Grubbs' test excluded from means and SEM)

Table S15 Best fit parameters for the data shown in Figure 4 obtained using a 4-parameter logistic function. Values in parentheses represent the standard errors of the fitted parameters.

Parameter	Hamster – *L. infantum*	Mouse – *L. infantum*
Bottom	0.99 (7.96)	0.51 (2.28)
Top	100 (15.8)	100 (3.20)
Slope	4.62 (5.53)	2.67 (0.58)
unbound ED_{50}	1.92 (0.27)	8.78 (0.58)
unbound ED_{95} [a]	3.6	27
r^2	0.910	0.995

[a] Estimated using the fitted parameters

Table S16 Input parameters for GastroPlus simulations

Parameter	Value
Human body weight (kg)	50
Log $D_{7.4}$	2.57
pKa	5.58
FaSSGF solubility (mg/mL)	1.19 (pH 1.6)
FaSSIF solubility (mg/mL)	0.011 (pH 6.5)
FeSSIF solubility (mg/mL)	0.054 (pH 5.8)
Effective human jejunal permeability (cm/s)	5.57
Plasma clearance (L/h)	3.08
Human fraction unbound	0.059
Human blood:plasma	0.8

Table S17 Early *in silico* and *in vitro* cardiotoxicity assessment of DNDI-6174

Platform	Assay	Result
in silico simulation (AP-preDICT)	Qube hERG	IC50 = 31.6 μM
	Qube NaV1.5	IC50 > 50 μM
	Qube CaV1.2	IC50 = 25.1 μM
	QPATCH CaV1.2	IC50 = 37.82 μM
in vitro	Qpatch hERG	3.81 % inhibition at 1 μM<br>48.01 % inhibition at 10 μM
in vitro	hIPSc-CM	No significant change in the field potential duration, the spike amplitude and the beat period throughout the concentration range tested (0.37, 1.11, 3.33, 10 and 30 μM).<br>No prediction of significant QT prolongation or significant change in cardiac conduction.

Table S18 Complete *in vitro* cytotoxicity profiling of DNDI-6174

Cell line		Result (CC_{50})
3T3	Mouse embryo fibroblast	> 37 µM
HEPG2	Human hepatocellular carcinoma	> 64 µM
HFF	Human foreskin fibroblast	> 64 µM
L-6	Rat skeletal myoblast	> 63 µM
MRC-5	Human lung fibroblast	> 64 µM
PMM	Primary mouse macrophage	> 64 µM
THP1	Human monocytic leukaemia	> 50 µM
U2OS	Human osteosarcoma	> 80 µM
Vero	Monkey kidney epithelia	> 50 µM

Each value is the mean of at least two independent assays, conducted in duplicate at each concentration.

Table S19 DNDI-6174 profile in a panel mammalian receptors, enzymes and ion channels

Receptor / enzyme / channel	Species	DNDI-6174 (at 10 µM) activity (% of DMSO control)
A2A	human	83.8
α1A	human	25.1
α2A	human	60.3
β1	human	112.9
β2	human	102.1
BZD (central)	rat	90.2
CB1	human	104.5
CB2	human	96.1
CCK1 (CCKA)	human	126.2
D1	human	63.1
D2S	human	77.6
ETA	human	102.6
H1	human	91.8
H2	human	120.3
MAO-A	rat	94.3
M1	human	91.5
M2	human	92.9
M3	human	98.5
N neuronal α4β2	human	100.2
δ (DOP)	human	101.7
κ (KOP)	rat	90.6
µ (MOP)	human	94.3
5-HT1A	human	106.7
5-HT1B	rat	91.4
5-HT2A	human	93.9
5-HT2B	human	81.4
5-HT3	human	104.8
GR	human	93
AR	human	99.1
V1 a	human	100.7
Ca2+ channel, (L,dihydropyridine site)	rat	89.6
Potassium Channel hERG (human)- [3H] Dofetilide	human	94.2
KV channel	rat	98.7
Na+ channel (site 2)	rat	73.9
norepinephrine transporter	human	81.9
dopamine transporter	human	60.6
5-HT transporter	human	69.9
Lck kinase	human	100.5
COX1	human	115.3
COX2	human	137.1

PDE3A	human	30.1
PDE4D2	human	27.1
acetylcholinesterase	human	96.8
NMDA receptor	rat	89.6

Table S20 Assessment of human complex III activity and mitochondrial toxicity.

	DNDI-6174
Human complex III	<20% inhibition at 200µM
Calcium loading capacity, HEK mitochondria	Mean pXC50 = 5 (10 µM)
MitoXpress	HepG2 cells: Oxygen consumption reduced at concentrations ≥ 66 µM up to maximum reduction of 28% at 200 µM<br><br>THP-1: pXC50 <4.3 (>50 µM)
Seahorse MST (HepG2 cells)	OCR Basal: MEC = 95.6 µM (maximum response = 14% at 200µM). AC_{50} >200 µM<br><br>No significant response observed on reserve capacity, ECAR, maximum capacity or ATP production<br><br>ECAR: transient increase observed at 200 µM

Human complex III EC50 values are the mean of at least two biological replicates consisting of at least two technical replicates (n ≥ 2).
MEC= Minimum Effective Concentration, was defined as the concentration that significantly crosses vehicle control threshold.

Table S21 Cytochrome P450 inhibition by DNDI-6174

CYP isoform	Direct inhibition IC_{50} (µM)	Time-dependent inhibition IC_{50} (µM) - without NADPH / with NADPH preincubation
CYP1A2	5.1	3.4 / 1.0
CYP2B6	>20	>20 / >20
CYP2C8	>20	>20 / 3.2
CYP2C9	14.9	13.8 / 16.4
CYP2C19	19.0	13.6 / 7.4
CYP2D6	19.0	9.8 / 0.49
CYP3A45	MID: >20	MID: >20 / 1.9
	TST: 12.3	TST: 8 / 0.71

MID = midazolam; TST = testosterone
Bold values indicate a significant shift (>4-fold) to a lower IC_{50} with preincubation in the presence of NADPH

References

1. S. Hendrickx, G. Caljon, L. Maes, *In vitro* growth inhibition assays of *Leishmania spp*. *Methods Mol Biol* **2116**, 791-800 (2020).
2. S. Hendrickx, J. Beyers, A. Mondelaers, E. Eberhardt, L. Lachaud, P. Delputte, P. Cos, L. Maes, Evidence of a drug-specific impact of experimentally selected paromomycin and miltefosine resistance on parasite fitness in *Leishmania infantum*. *J Antimicrob Chemother* **71**, 1914-1921 (2016).
3. S. Hendrickx, A. Mondelaers, E. Eberhardt, L. Lachaud, P. Delputte, P. Cos, L. Maes, Intracellular amastigote replication may not be required for successful in vitro selection of miltefosine resistance in *Leishmania infantum*. *Parasitol Res* **114**, 2561-2565 (2015).
